# A Windmill-Shaped Molecule with Anthryl Blades to Form Smooth Hole-Transport Layers via a Photoprecursor Approach

**DOI:** 10.3390/ma13102316

**Published:** 2020-05-18

**Authors:** Akihiro Maeda, Aki Nakauchi, Yusuke Shimizu, Kengo Terai, Shuhei Sugii, Hironobu Hayashi, Naoki Aratani, Mitsuharu Suzuki, Hiroko Yamada

**Affiliations:** 1 Division of Materials Science, Graduate School of Science and Technology, Nara Institute of Science and Technology (NAIST), 8916-5 Takayama-cho, Ikoma, Nara 630-0192, Japan; wsgtx310@gmail.com (A.M.); masquarade.ribbon@icloud.com (A.N.); orgvy3s92@gmail.com (Y.S.); me1307kengo@gmail.com (K.T.); sshuhei.0627@gmail.com (S.S.); hhayashi@ms.naist.jp (H.H.); aratani@ms.naist.jp (N.A.); 2 Division of Applied Chemistry, Graduate School of Engineering, Osaka University, 2-1 Yamadaoka, Suita, Osaka 565-0871, Japan

**Keywords:** organic photovoltaics, morphology, layer-by-layer solution process, acenes

## Abstract

Preparation of high-performance organic semiconductor devices requires precise control over the active-layer structure. To this end, we are working on the controlled deposition of small-molecule semiconductors through a photoprecursor approach wherein a soluble precursor compound is processed into a thin-film form and then converted to a target semiconductor by light irradiation. This approach can be applied to layer-by-layer solution deposition, enabling the preparation of p–i–n-type photovoltaic active layers by wet processing. However, molecular design principles are yet to be established toward obtaining desirable thin-film morphology via this unconventional method. Herein, we evaluate a new windmill-shaped molecule with anthryl blades, 1,3,5-tris(5-(anthracen-2-yl)thiophen-2-yl)benzene, which is designed to deposit via the photoprecursor approach for use as the p-sublayer in p–i–n-type organic photovoltaic devices (OPVs). The new compound is superior to the corresponding precedent p-sublayer materials in terms of forming smooth and homogeneous films, thereby leading to improved performance of p–i–n OPVs. Overall, this work demonstrates the effectiveness of the windmill-type architecture in preparing high-quality semiconducting thin films through the photoprecursor approach.

## 1. Introduction

The control of active-layer structures is a major concern in preparing organic electronic devices. For instance, achievement of high-performance organic photovoltaic devices (OPVs) largely rests on the formation of adequate active-layer morphology for generating and collecting charge carriers [1,2,3,4]. Likewise, efficient electricity-to-light conversion in organic light-emitting diodes comes out only when multiple materials are stacked in a specific sequence with the morphology of each layer carefully optimized [5,6]. Accordingly, the material design for organic electronic devices is more than just tuning of electronic characteristics at the molecular level. Rather, the design of high-performance materials requires careful considerations of morphology-related factors such as crystallinity and miscibility [7,8,9,10]. On the same basis, there are increasing demands toward reliable and versatile methods for controlled preparation of active layers [11,12,13,14].

**Figure 1:**
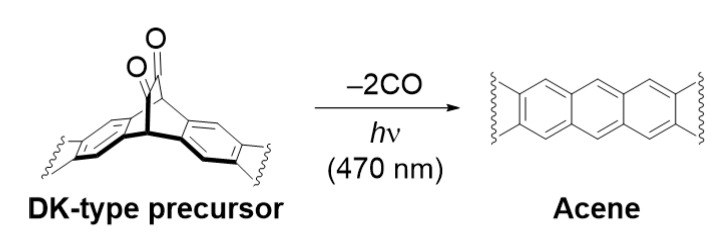
Decarbonylative aromatization of DK-type photoprecursors to form acenes. This reaction is typically induced by visible light about 470 nm which corresponds to the electronic transition from the n-orbital to π-antibonding orbital of the DK unit.

In the above context, we are working on the solution deposition of acene-based small-molecule semiconductors via a “photoprecursor approach” as a means to prepare active layers of organic electronic devices [15,16,17,18]. This approach relies on the photo-induced decarbonylative aromatization of α-diketone (DK)-type precursors (Figure 1), the mechanism of which has been discussed previously [18]. Specifically, well-soluble DK-type derivatives are solution-deposited, and then irradiated typically with visible light of around 470 nm to generate corresponding acene compounds. The DK-to-acene conversion is usually accompanied by a decrease in solubility, and when the solubility of the resulting acene is sufficiently low, another material can be deposited on top of it as a solution. Accordingly, the photoprecursor approach enables controlled layer-by-layer solution deposition of organic semiconductors.

The photoprecursor approach has been applied to the preparation of OPVs having p–i–n-type active layers, in which a p:n intermixed layer (i-sublayer) is sandwiched between neat p- and n-type materials (p- and n-sublayers) [19,20,21]. This triple-layer structure is expected to bring about the combination of efficient charge-carrier generation in the i-sublayer and selective, swift charge-carrier extraction through the p- and n-sublayers, thereby benefitting the photovoltaic process [22,23,24,25]. In addition, p- and n-sublayers can serve as exciton-blocking layers upon proper choice of materials. The maximum benefit of this structure can be gained by optimizing each sublayer in terms of material, morphology, and thickness. As a proof of this concept, we have demonstrated that a ternary p–i–n OPV can achieve a power-conversion efficiency (PCE) almost twice as high as the corresponding simple bulk-heterojunction (BHJ) device (5.9 vs. 3.0%) [20].

**Figure 2:**
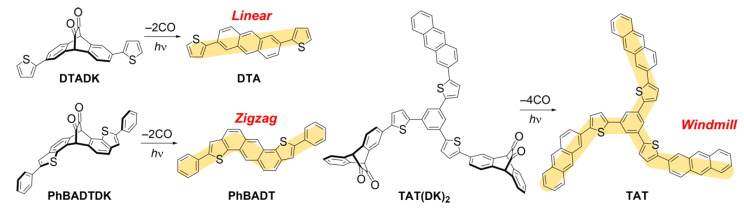
Chemical structures of the hole-transport compounds and the corresponding DK-type photoprecursors compared in this work as p-sublayer materials in p–i–n-type OPVs. The linear-shaped DTA and zigzag-shaped PhBADT have been employed in our previous studies [19,20,21], while the windmill-shaped TAT is newly designed.

One of the remaining challenges with the photoprecursor approach is establishing molecular-design principles for highly effective, robust p-sublayer (or hole-transport) materials. The compounds employed in our previous studies (2,6-di(thiophen-2-yl)anthracene (DTA) and 2,8-diphenylanthra[1,2-*b*:5,6-*b*’]dithiophene (PhBADT), Figure 2) are not ideal in that they are strongly aggregating and form highly rough surfaces [19,20,21]. Indeed, the roughness was too high to determine the effective film thickness, and thus it was not possible to estimate hole mobilities in those materials by the space-charge-limited-current (SCLC) method. The high inhomogeneity of the films also prohibited fine-tuning of their thicknesses.

With these in mind, we have newly designed a windmill-shaped molecule, 1,3,5-tris(5-(anthracen-2-yl)thiophen-2-yl)benzene (TAT), as a p-sublayer material for solution-processed p–i–n-type photovoltaic active layers (Figure 2). Molecules with a branched backbone, including windmill-shaped ones, are known to form amorphous, smooth films, and to show decent charge-carrier mobilities upon proper molecular design. As such, branched molecular structures have been continuously featured in the design of organic semiconductors [26,27,28,29,30]. On the other hand, morphological behaviour of molecules in the deposition by the photoprecursor approach is often dissimilar from that in conventional techniques, largely because of the change in molecular structure during the solid-state photoreaction [16,17]. In addition, the layer-by-layer solution process requires sufficient insolubility of post-deposition materials in order to avoid undesired dissolution or interlayer mixing. The following sections experimentally examine these aspects of TAT, as well as its semiconducting properties. The results show that the windmill-shaped molecule is better qualified for p-sublayer in solution-processed p–i–n active layers as compared to the previously employed linear-shaped DTA and zigzag-shaped PhBADT.

## 2. Results and Discussion

### 2.1. Molecular Design

High transparency and hole mobility are both key requirements for an ideal p-sublayer in p–i–n-type OPVs. Transparency across a wide range of wavelengths is particularly important for devices with the conventional (i.e., non-inverted) geometry to allow incident sunlight to reach and be absorbed by the i-sublayer. In this regard, the central benzene ring of TAT is substituted at the 1,3,5-positions in order to minimize the bathochromic shift of absorption onset caused by the orbital delocalization between substituents. Density functional theory (DFT) calculations at the B3LYP/6-31G(d) level have predicted that TAT is similar to the two previous p-sublayer materials in terms of the difference between the highest occupied molecular orbital (HOMO) and the lowest unoccupied molecular orbital (LUMO) energies: 3.21, 3.18, and 3.29 eV for TAT, DTA, and PhBADT, respectively (Figure S1a in Supplementary Material). Additionally, the optimized conformation of TAT is mostly planar with relatively small torsion angles of 20–30° (Figure S1b and Table S1), owing to the structure of not having the *ortho*-substitution pattern at the central benzene ring, while having a five-membered thienylene spacer between the benzene core and anthracene blades. The high planarity is favorable for forming intermolecular π–π contacts and thus carrier-transport paths in the solid state.

For layer-by-layer solution deposition, the materials should possess a high-enough solubility in the precursor form, and at the same time a low-enough solubility after photoreaction. In this regard, the precursor TAT(DK)_2_ is equipped with two solubilizing DK units (Figure 2). This is because one DK unit is not enough to guarantee TAT an adequate solubility for solution processing. On the other hand, introduction of three DK units has turned out to considerably increase the synthetic and purification costs because of, for example, a solubility issue or severe trailing on silica gel. Importantly, the photoreaction product TAT is essentially insoluble to the cast solvent (chloroform) as shown below.

### 2.2. Synthesis and Photoreaction of the Precursor

**Scheme 1:**
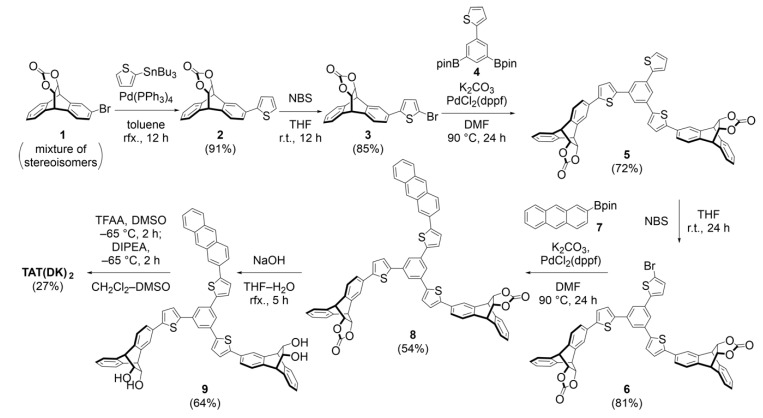
Synthetic route to TAT(DK)_2_. NBS: *N*-bromosuccinimide, THF: tetrahydrofuran, Bpin: pinacolatoboryl, dppf: 1,1′-bis(diphenylphosphino)ferrocene, DMF: *N*,*N*-dimethylformamide, TFAA: trifluoroacetic anhydride, DMSO: dimethyl sulfoxide, DIPEA: *N*,*N*-diisopropylethylamine. All products were isolated as a mixture of stereoisomers.

As summarized in Scheme 1, the synthesis of the photoprecursor TAT(DK)_2_ was started from bromoanthracene–vinylene carbonate adduct **1** [31]. This Diels–Alder adduct was subjected to the Stille coupling with 2-(tributylstannyl)thiophene to form compound **2** followed by bromination at the α-position of the thienyl group. The resulting bromide **3** was then reacted with diboronate **4** under Suzuki cross-coupling conditions to form **5** having the 1,3,5-trisubstituted benzene core. Another bromination was performed to afford compound **6**, which was then subjected to the Suzuki coupling with 2-borylanthracene **7** to construct the windmill-like backbone of compound **8**. After saponification of the carbonate units, bis(diol) **9** was oxidised under Swern conditions to give the target compound TAT(DK)_2_ in 27% yield. Note here that the reaction yield of TAT(DK)_2_ is almost quantitative as judged from nuclear magnetic resonance (NMR) analysis of a crude product; however, the removal of minor by-products required repetitive column chromatography and reprecipitation treatments, limiting the isolation yield of a high-purity portion. This inefficiency in purification is a target of improvement in future work. Details of the synthetic procedures and spectroscopic data are provided in Section 4.2 and Figures S2–S16.

**Figure 3:**
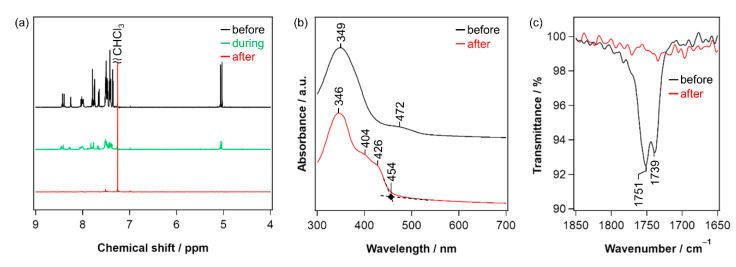
Spectroscopic changes associated with the photoreaction of TAT(DK)_2_: (**a**) ^1^H NMR spectra of a CDCl_3_ solution (400 MHz); (**b**) UV–vis absorption spectra of a thin film; (**c**) IR spectra of a thin film.

The light-induced decarbonylation of TAT(DK)_2_ in a chloroform solution was monitored by ^1^H NMR. As shown in Figure 3a, irradiation with visible light at 470 nm resulted in gradual decrease and eventual disappearance of all the signals except the residual solvent peak, with the concomitant formation of a yellow precipitate. This observation is in accordance with the expected lowering in solubility upon the conversion to TAT. At the same time, the insolubility of TAT prevents direct confirmation of its formation; however, it would be still safe to assume its formation considering previous examples in which quantitative conversion of DK-type precursors to the corresponding anthracene and even pentacene derivatives were confirmed by NMR [31,32,33]. The complete disappearance of the signals indicates that TAT is quite insoluble in chloroform, the cast solvent employed in this work, and thus solution deposition of another material on top of TAT should be possible to construct the p–i–n structure. As an additional note, TAT(DK)_2_ is well soluble in a variety of common organic solvents including toluene, dichloromethane, and tetrahydrofuran, while TAT is essentially insoluble in these solvents.

The intended photoreaction proceeded smoothly also in the thin-film state. Figure 3b compares the ultraviolet–visible (UV–vis) absorption spectra of thin films before and after light irradiation. The TAT(DK)_2_ film showed a broad, relatively weak absorption around 470 nm, which can be assigned to the n–π* transition of the DK moiety [18]. This broad peak disappeared upon irradiation, while absorption around 370–440 nm was enhanced with the formation of shoulders at 404 and 426 nm. These changes can be explained by the progress of the light-induced decarbonylation to generate the anthryl group. In addition, infrared (IR) spectroscopy confirmed the decarbonylation in the thin-film state as shown in Figure 3c, in which the C=O stretch bands at 1739 and 1751 cm^−1^ fully disappeared after irradiation. Note that the possibility would be low for the anthracene moieties of TAT to undergo the light-induced dimerization, because the irradiation wavelength (about 470 nm) is longer than the absorption band edge of TAT (454 nm). In addition, such a dimerization in the solid state requires a specific arrangement of neighbouring molecules, the occurrence of which should be rare in amorphous films (see the next section).

### 2.3. Thin-Film Characteristics

**Figure 4:**
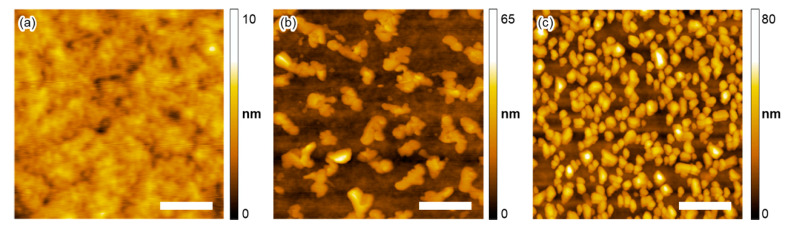
Tapping-mode atomic-force-microscopy images of thin films prepared through the photoprecursor approach: (**a**) TAT (*R*_RMS_ = 0.9 nm); (**b**) DTA (7.0 nm); (**c**) PhBADT (11.9 nm). The scale bars correspond to 0.5 μm.

**Figure 5:**
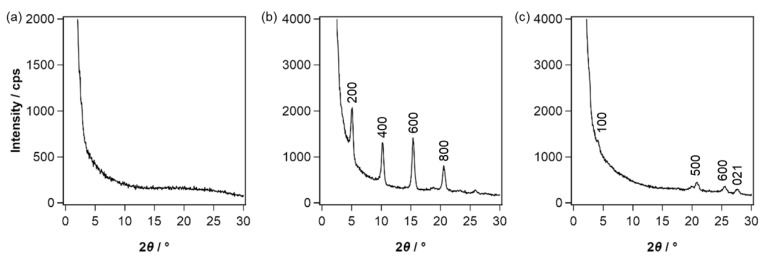
Out-of-plane X-ray-diffraction profiles of thin films prepared through the photoprecursor approach: (**a**) TAT; (**b**) DTA; (**c**) PhBADT.

Smoothness and homogeneity are of high importance for semiconducting thin films in terms of minimizing defects and controlling the thickness. In this regard, thin films of TAT, DTA, and PhBADT were probed with an atomic force microscope (AFM) for evaluating their surface topology. The thin films were prepared through the photoprecursor approach on indium–tin-oxide (ITO) substrate coated with poly(3,4-ethylenedioxythiophene) polystyrene sulfonate (PEDOT:PSS) in the same manner as the preparation of p-sublayers for the p–i–n-type OPVs discussed in the next section (see Section 4.4 for detailed procedure). As shown in Figure 4, TAT formed a highly smooth and homogeneous surface with a root-mean-square roughness (*R*_RMS_) of only 0.9 nm. On the other hand, the precedent materials DTA and PhBADT gave inhomogeneous films containing large grains of up to a few hundred nanometres in lateral dimension, with *R*_RMS_ as high as 7.0 and 11.9 nm, respectively (see Figure S17 for additional images of TAT and PhBADT, or previous papers for DTA [16,21]). Therefore, it is assumed that TAT should have a significantly lower tendency to aggregate during the solid-state photoreaction in comparison with the other two compounds (note here that DK-type precursors generally form smooth, homogeneous films). This is supported by the out-of-plane X-ray diffraction data of the thin films (Figure 5), in which TAT showed no recognizable diffraction peak, while the other two showed multiple peaks that could be assigned to crystal planes of the corresponding single-crystal X-ray structures [34,35]. The amorphousness of TAT is reasonable if one considers its large conformational flexibility originating from rotation of the six single bonds, in addition to the general low crystallinity of branched molecules [26,27,28,29,30].

**Figure 6:**
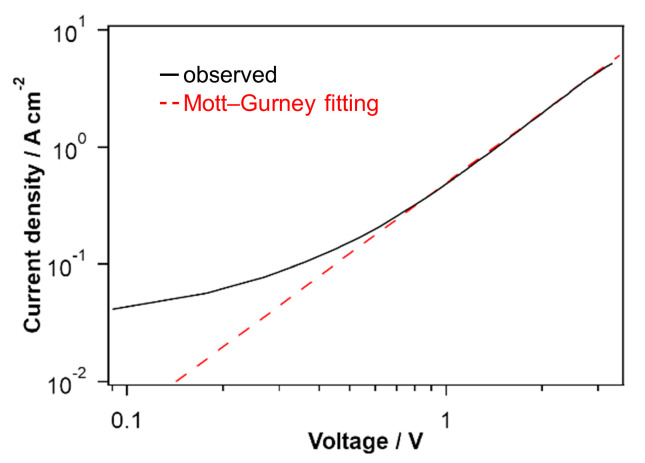
Current–voltage characteristics of a hole-only device with a structure of (ITO/MoO_3_/TAT/MoO_3_/Al). The *μ*_h_ was estimated as 1.4 × 10^−4^ cm^2^ V^−1^ s^−1^ based on the Mott–Gurney law. Voltage for the observed data is defined as *V*_appl_–*V*_bi_–*V*_s_, wherein *V*_appl_ is the applied voltage, *V*_bi_ is the estimated built-in voltage, and *V*_s_ is the estimated voltage drop associated with series resistance.

The smoothness of the TAT film enabled us to define the effective thickness and thus to estimate charge-carrier mobility by the SCLC method. The hole mobility (*μ*_h_) in a TAT film was estimated as 1.4 × 10^−4^ cm^2^ V^−1^ s^−1^ (Figure 6), which is within the typical range for hole-transporting small-molecule materials (10^−4^–10^−6^) [36,37,38]. In the context of using this material as the p-sublayer of p–i–n-type OPVs, its *μ*_h_ should be at least the same level as that of the donor material in the i-sublayer for assuring efficient hole extraction. The donor material employed in this study is 2,6-di[5′-(2-ethylhexyl)-2,2′-bithien-5-yl]anthracene (EBDBTA), shown in the next section, and its *μ*_h_ has been estimated to be 6.4 × 10^−5^ cm^2^ V^−1^ s^−1^ (Figure S18). While determination of exact *μ*_h_ values in these materials is beyond the scope of this contribution, it would be safe to expect based on the estimated values that TAT does not interfere the hole transport toward the anode in OPVs. It should be also noted that the TAT film has the absorption band edge at 454 nm (Figure 3b), being transparent across most of the visible range. Thus, TAT is considered as a promising candidate for the p-sublayer in terms of morphology, charge-carrier mobility, and transparency.

### 2.4. Photovoltaic Performance

**Figure 7:**
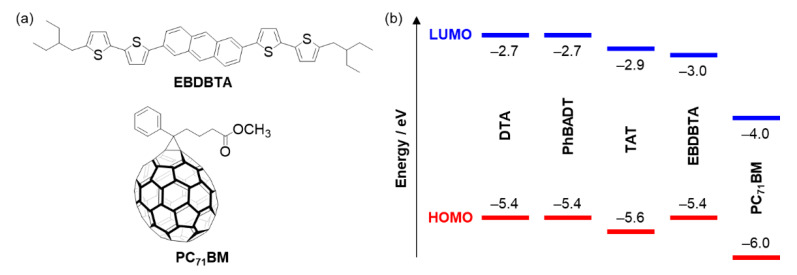
(**a**) Chemical structures of EBDBTA and PC_71_BM. (**b**) Frontier-orbital energy levels of the active-layer materials in the thin-film state. For DTA, PhBADT, TAT, and EBDBTA, the HOMO levels are the IE measured by photoelectron spectroscopy in air, while the LUMO levels are the addition of HOMO and optical band-gap energies. The values for DTA, PhBADT, EBDBTA, and PC_71_BM are adopted from the literature [19,20,39]. The photoelectron spectrum of TAT is shown in Figure S19.

The performance of TAT was evaluated in p–i–n-type OPVs having an active-layer structure of (TAT/EBDBTA:PC_71_BM/PC_71_BM) (PC_71_BM: [6,6]-phenyl-C_71_-butyric acid methyl ester; Figure 7a). The donor compound in the i-sublayer, EBDBTA [19], is the material of choice because of its synthetic accessibility and consistency in performance. Regarding the alignment of orbital energy levels, the experimentally determined HOMO level of TAT is slightly lower than that of EBDBTA (−5.6 vs. −5.4 eV, Figure 7b) (hereafter the term “molecular orbital” is applied to not only isolated, but also condensed molecules in the thin-film state. The HOMO energy in the thin-films state is defined as the ionization energy (IE) measured by photoelectron spectroscopy in air). Although this situation looks unideal for hole extraction, our previous results have indicated that such a rather minor misalignment would not harm the photocurrent generation in p–i–n-type OPVs [19]. The LUMO energy level of TAT is higher than that of EBDBTA (−2.9 vs. −3.0 eV), owing to the larger HOMO–LUMO difference (i.e., optical band gap) for the former (herein the LUMO energy is defined as “HOMO energy + optical band-gap energy”). This is favorable for avoiding the nonproductive electron transfer from EBDBTA domains to the p-sublayer and consequential charge recombination. Not surprisingly, the HOMO and LUMO energy levels of TAT are considerably higher than those of PC_71_BM (HOMO: −6.0 eV, LUMO: −4.0 eV [39]) ensuring the intended directional electron transport from the p- to n-sublayer.

The p–i–n-type OPVs were fabricated on ITO-glass substrates with a buffer layer of PEDOT:PSS (poly(3,4-ethylenedioxythiophene):polystyrene sulfonate). The p-sublayer was prepared by spin-coating a 0.5 mg mL^−1^ solution of TATDK in chloroform, followed by irradiation with a blue light emitting diode (LED) at 400 mW cm^−2^ for 30 min, creating a TAT film of ca. 9-nm in thickness as determined by a stylus surface profiler. The i- and n-sublayers were prepared in accordance with a previous paper [19], on which a fullerene-based cathode buffer (ETL-1 [40]) and aluminum cathode were sequentially deposited to complete the device structure. For comparison, OPVs comprising DTA or PhBDTA as the p-sublayer material were also prepared in the same manner. Details of the device preparation process are described in Section 4.4.

As summarized in Table 1, TAT afforded somewhat higher PCEs than the other two p-sublayer materials: the best (average) PCEs are 3.24 (3.11), 3.13 (3.03), and 3.12 (2.93)% for the devices with TAT, DTA, and PhBDTA, respectively. The higher efficiency with TAT can be ascribed to the superiority in open-circuit voltage (*V*_OC_) and short-circuit current density (*J*_SC_). The *V*_OC_ of the best TAT-device is 0.899 V, while those of the DTA- and PhBADT-devices are 0.877 and 0.876 V. This relatively minor increase of about 20 mV with TAT does not reflect the substantial difference of 0.2 eV in the HOMO level (Figure 7b). Thus, it is assumed that the HOMO level of p-sublayer does not directly affect the *V*_OC_ of the present devices. The increased *V*_OC_ of the TAT-device may be rationalized by other possibilities such as reduction of charge recombination owing to improvement in hole-extraction efficiency [41]. The reduction of charge recombination should also increase *J*_SC_, which was actually observed for the TAT-devices (6.44 mA cm^−2^ at best) in comparison with the DTA- and PhBDTA-devices (6.23 mA cm^−2^ at best for both). The difference in *J*_SC_ may also be related to the exciton quenching by the PEDOT:PSS buffer; namely, the better surface coverage with TAT leads to a lower chance of direct contact of PEDOT:PSS with the i-sublayer, thereby reducing exciton quenching. The exciton quenching by PEDOT:PSS has been invoked previously as a limiting factor of OPV performance [42]. It is also worth pointing out that the lower HOMO level of TAT than EBDBTA (−5.6 vs. −5.4 V, Figure 7b) does not cause apparent decrease in *J*_SC_ and FF, which is in accordance with the previous observation mentioned above.

**Figure 8:**
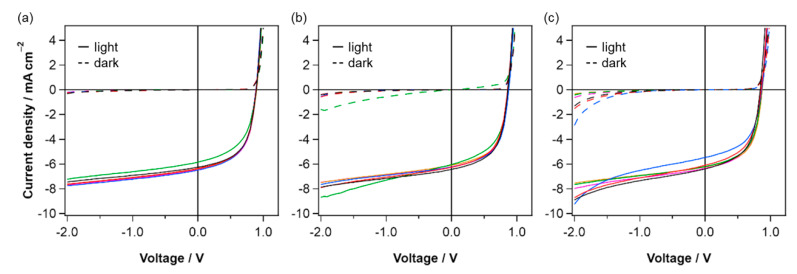
*J*–*V* curves of the p–i–n-type OPVs comprising different p-sublayer materials: (**a**) TAT; (**b**) DTA; (**c**) PhBADT. Data of six devices are overlaid in each graph. The data are those used to calculate the average photovoltaic parameters in Table 1.

The superiority of TAT has become evident also from the smaller leakage current. Specifically, the dark current at a bias voltage of −2 V (*J*_dark_(−2)) is significantly lower with TAT than DTA and PhBADT (0.15, 0.43, and 0.46 mA cm^−2^ on average, respectively) (Table 1, Figure 8 and Figure S20). The AFM images in Figure 4 show that the aggregates of DTA and PhBADT grow up to several tens of nanometers in height. This may cause considerable uncertainty in film thickness and material distribution within the active layer, thereby lowering the reproducibility of OPV performance. High roughness of the p-layer can also increase the possibility for formation of structural defects in the active layer, which most likely leads to, for example, higher probability of current leakage and thus lower reproducibility of OPVs. Interestingly, at the same time, inhomogeneity of the p-sublayer is not detrimental to the photovoltaic performance as can be seen in the essentially unchanged FF among the three cases. This aspect will be examined in detail and reported in due course.

## 3. Concluding Remarks

To summarize, this work has demonstrated the effectiveness of the windmill-like molecular architecture in forming highly homogeneous thin films through the photoprecursor approach. The newly designed p-sublayer (or hole-transport) material, TAT, enabled the preparation of p–i–n-type OPVs with improved performance and a smaller leakage current as compared to the precedent materials. While it has been widely known that branched molecules can form highly-performing carrier-transport layers by conventional deposition processes, the present contribution is the first experimental confirmation that such a molecular design is highly practical in the layer-by-layer deposition via the photoprecursor approach. Considering the unique features and wide applicability of the photoprecursor approach [21], the above results are expected to serve as a basis for designing a new class of small-molecule carrier-transport materials.

## 4. Materials and Methods

### 4.1. General

All reactions were carried out under argon unless otherwise noted. “Room temperature” (r.t.) means 15–25 °C. Solvents and chemical reagents for synthesis were reagent grade obtained from commercial sources and used without further purification. Compound **1** [31], tributyl(thiophen-2-yl)stannane [43], 2-(3,5-dibromophenyl)thiophene [44], compound **7** [31] were prepared as described in literature. Flash column chromatography was performed on silica gel purchased from Kanto Chemical (Silica Gel 60N, 60 Å, 40–50 nm). Analytical thin-layer chromatography (TLC) was conducted on Merck 200-nm thickness silica-gel plates with a fluorescent indicator (1.05554.0001). Visualization of TLC was accomplished with UV light at 254 nm. ^1^H NMR and ^13^C{^1^H} NMR spectra were recorded on a JEOL JNM-ECX 400P spectrometer (400 MHz) at 294 K using tetramethylsilane as internal standard. High-resolution mass spectra were measured on a JEOL AccuTOF/JMS-T100LC (electrospray ionization, ESI), JEOL JMS-700 (electron ionization, EI), or JEOL SpiralTOF/JMS-S3000 (matrix-assisted laser desorption/ionization–time of flight, MALDI–TOF) mass spectrometer. DFT calculations were performed using the Gaussian 09 program suite [45] at the B3LYP/6-31G(d) level of theory. For the monitoring by ^1^H NMR, approximately 1 mg of a photoprecursor was dissolved in 0.5 mL of CDCl_3_ in an NMR tube equipped with a J. Young valve. The solution was degassed by three freeze–pump–thaw cycles, and the tube was refilled with argon. The sample solution was irradiated with a xenon lamp (NIPPON P·I PCS-MH375RC, ~450 W) through a blue-cut filter at a distance of about 5 cm, and periodically subjected to ^1^H NMR measurement until no change was observed (about 20 min in total).

### 4.2. Chemical Synthesis and Spectroscopic Data

#### 4.2.1. 2-(Thiophen-2-yl)-9,10-dihydro-9,10-[4,5]epidioxoloanthracen-13-one (**2**)

In a 100 mL two-necked round-bottom flask equipped with a condenser, bromide **1** (2.3 g, 6.7 mmol), tributyl(thiophen-2-yl)stannane (3.4 mL, 4.0 g (purity ~75%), 8.1 mmol, 1.2 equiv), and Pd(PPh_3_)_4_ (50 mg, 43 μmol, 0.64 mol%) were dissolved in toluene (25 mL) under argon. The solution was refluxed for 12 h, and the solvent was removed by evaporation in vacuo. The crude product was purified by flash column chromatography on silica gel with 10 wt% of K_2_CO_3_ (CH_2_Cl_2_/hexanes, 4:1) to give compound **2** as a pale yellow solid (2.1 g, 6.1 mmol, 91%). The product was obtained as a mixture of stereoisomers regarding the orientation of the carbonate unit. ^1^H NMR (400 MHz, CDCl_3_) δ: 7.62–7.61 (1H), 7.50–7.45 (1H), 7.41–7.36 (3H), 7.31–7.19 (4H*), 7.08–7.04 (1H), 4.73–4.65 (2H), 4.92–4.84 (2H) (multiplicities and coupling constants could not be determined because of severe overlapping of isomer peaks; *overlapped with CHCl_3_); ^13^C{^1^H} NMR (101 MHz, CDCl_3_) δ: 154.09, 154.00, 153.83, 143.76, 143.43, 139.56, 138.47, 138.26, 137.67, 137.52, 137.44, 137.29, 137.11, 136.92, 136.74, 136.23, 136.17, 136.05, 135.96, 135.85, 135.48, 135.44, 134.19, 133.89, 128.14, 128.06, 127.99, 127.93, 127.89, 127.79, 127.75, 127.65, 127.03, 126.87, 126.82, 126.71, 126.66, 126.58, 126.11, 125.98, 125.77, 125.71, 125.64, 125.28, 125.19, 124.95, 124.12, 123.50, 123.42, 123.24, 76.23, 76.15, 76.01, 75.94, 75.90, 75.85, 47.75, 47.68, 47.48, 47.46, 47.41, 47.37, 47.17, 47.14 (overlapping of signals is expected between stereoisomers); HRMS (ESI) *m*/*z*: calcd for C_21_H_14_BrNaO_3_S^+^ ([M + Na]^+^), 369.0561; found, 369.0561.

#### 4.2.2. 2-(5-Bromothiophen-2-yl)-9,10-dihydro-9,10-[4,5]epidioxoloanthracen-13-one (**3**)

In a 200 mL three-necked round-bottom flask, compound **2** (2.50 g, 7.22 mmol) was dissolved in THF (50 mL) in air. *N*-bromosuccinimide (NBS) (1.40 g, 7.86 mmol) was added to the solution at 0 °C, and the solution was stirred at r.t. for 12 h in the dark. The reaction mixture was partitioned between CH_2_Cl_2_ (100 mL) and water (100 mL). The organic layer was isolated, washed with brine (100 mL), dried over Na_2_SO_4_, and evaporated in vacuo. The residue was purified by column chromatography on silica gel (CH_2_Cl_2_/hexanes, 4:1) to give compound **3** as a pale yellow solid (2.60 g, 6.11 mmol, 85%). The product was obtained as a mixture of stereoisomers regarding the orientation of the carbonate units. ^1^H NMR (400 MHz, CDCl_3_) δ: 7.52 (s, 1H), 7.41–7.37 (4H), 7.29–7.22 (2H*), 7.02–6.99 (2H), 4.92–4.87 (2H), 4.73–4.70 (2H) (multiplicities and coupling constants could not be determined because of severe overlapping of isomer peaks; *overlapped with CHCl_3_); ^13^C{^1^H} NMR (101 MHz, CDCl_3_) δ: 154.06, 153.95, 145.19, 144.86, 138.68, 137.36, 137.30, 137.26, 135.97, 135.92, 135.85, 133.38, 133.09, 130.99, 130.85, 128.01, 127.96, 127.87, 127.82, 127.66, 127.18, 126.69, 126.62, 126.26, 125.75, 125.67, 125.01, 124.90, 123.76, 123.69, 123.60, 122.89, 111.90, 111.55, 76.25, 76.18, 76.11, 76.07, 47.73, 47.40 (overlapping of signals is expected between stereoisomers); HRMS (ESI) *m*/*z*: calcd for C_21_H_13_BrNaO_3_S^+^ ([M + Na]^+^); 446.9667, found 466.9672.

#### 4.2.3. 2,2′-(5-(thiophen-2-yl)-1,3-phenylene)bis(4,4,5,5-tetramethyl-1,3,2-dioxaborolane) (**4**)

To a 100 mL two-necked round-bottom flask were added 2-(3,5-dibromophenyl)thiophene [44] (0.423 g, 1.33 mmol), bis(pinacolato)diboron (0.600 g, 2.36 mmol), KOAc (1.05 g, 10.7 mmol), and DMSO (30 mL). After addition of PdCl_2_(PPh_3_)_2_ (0.140 g, 0.199 mmol, 15 mol%), the mixture was deoxygenated by argon bubbling for 30 min. The reaction mixture was stirred at 80 °C for 24 h, cooled down to r.t., and partitioned between water (50 mL) and diethyl ether (50 mL). The organic layer was isolated, washed with brine (50 mL), dried over Na_2_SO_4_, filtered, and evaporated in vacuo. The residue was purified by flash column chromatography on silica gel (hexanes/AcOEt, 2:1) to give compound **4** as a colorless solid (0.277 g, 0.672 mmol, 51%). ^1^H NMR (400 MHz, CDCl_3_) δ: 8.19 (coursed t, 1H), 8.13 (d, *J* = 1.4 Hz, 2H), 7.40 (dd, *J* = 3.7, 0.9 Hz, 1H), 7.27 (dd, *J* = 5.0, 1.4 Hz, 1H), 7.06 (q, *J* = 2.9 Hz, 1H), 1.35 (s, 24H); ^13^C{^1^H} NMR (101 MHz, CDCl_3_) δ: 144.30, 140.30, 134.99, 133.14, 127.82, 124.68, 123.36, 83.89, 24.88; HRMS (EI) *m*/*z* calcd for C_22_H_30_B_2_O_4_S^+^ (M^+^), 412.2051; found, 412.2055.

#### 4.2.4. 2,2′-((5-(thiophen-2-yl)-1,3-phenylene)bis(thiophene-5,2-diyl))bis(9,10-dihydro-9,10-[4,5]epidi- oxoloanthracen-13-one) (**5**)

Bromide **3** (305 mg, 0.717 mmol) and boronate ester **4** (153 mg, 0.371 mmol), K_2_CO_3_ (479 mg, 3.46 mmol), and DMF (15 mL) were added to a 100 mL round-bottom flask. The mixture was deoxygenated by argon bubbling for 30 min, to which PdCl_2_(dppf)·CH_2_Cl_2_ (54.4 mg, 12 mmol%). The reaction mixture was stirred at 90 °C for 24 h before poured to water (200 mL). The mixture was extracted with AcOEt (100 mL), and the organic layer was washed with water (3 × 20 mL), dried over Na_2_SO_4_, filtered, and evaporated. The residue was purified by flash column chromatography on silica gel (CH_2_Cl_2_) and GPC (CHCl_3_) to afford compound **5** as a pale yellow amorphous solid (226 mg, 0.266 mmol, 72%). The product was obtained as a mixture of stereoisomers regarding the orientation of the carbonate units. ^1^H NMR (400 MHz, CDCl_3_) δ: 7.75–7.73 (3H), 7.68 (br s, 2H), 7.56–7.49 (2H), 7.44–7.35 (9H), 7.32–7.24 (7H*), 7.15–7.11 (1H), 4.97–4.91 (4H), 4.81–4.73 (4H) (multiplicities and coupling constants could not be determined because of severe overlapping of isomer peaks; *overlapped with CHCl_3_); ^13^C{^1^H} NMR (101 MHz, CDCl_3_) δ: 154.15, 154.09, 154.00, 153.97, 143.68, 143.58, 143.46, 143.36, 143.27, 143.15, 143.09, 142.98, 142.73, 142.61, 138.62, 138.58, 137.61, 137.49, 137.46, 137.43, 137.39, 137.24, 137.21, 137.03, 136.95, 136.88, 135.98, 135.92, 135.89, 135.87, 135.77, 135.72, 135.67, 135.58, 135.50, 135.41, 135.33, 133.97, 133.91, 133.64, 128.19, 128.15, 128.12, 128.01, 127.97, 127.89, 127.84, 127.15, 126.69, 126.65, 126.22, 126.21, 125.75, 125.69, 125.59, 125.51, 125.42, 125.36, 125.29, 125.05, 124.99, 124.87, 124.83, 124.80, 124.76, 124.47, 124.40, 123.96, 123.94, 123.87, 123.82, 122.95, 122.44, 122.28, 122.20, 121.94, 121.88, 76.30, 76.24, 76.17, 76.14, 47.89, 47.83, 47.47, 47.44 (overlapping of signals is expected between stereoisomers); HRMS (MALDI–TOF, DCTB) *m*/*z* calcd for C_52_H_32_O_6_S_3_ 848.1356 (M^+^); found, 848.1275.

#### 4.2.5. 2,2′-((5-(5-bromothiophen-2-yl)-1,3-phenylene)bis(thiophene-5,2-diyl))bis(9,10-dihydro-9,10- [4,5]epidioxoloanthracen-13-one) (**6**)

Compound **5** (203 mg, 0.239 mmol) was dissolved in THF (35 mL) in air. A separately prepared solution of NBS (62.4 mg, 0.351 mmol) in THF (25 mL) was added slowly to the solution of compound **6** at 0 °C in the dark. The mixture was stirred at r.t. for 24 h in the dark before partitioned between CH_2_Cl_2_ (80 mL) and H_2_O (80 mL). The organic layer was isolated, washed with H_2_O (3 × 20 mL), dried over Na_2_SO_4_, filtered, and evaporated in vacuo. The residue was purified by flash column chromatography on silica gel (CH_2_Cl_2_/MeOH, 20:1) to give compound **6** as a colorless solid (179 mg, 0.193 mmol, 81%). The product was obtained as a mixture of stereoisomers regarding the orientation of the carbonate units. ^1^H NMR (400 MHz, CDCl_3_) δ: 7.76–7.75 (1H), 7.69 (br s, 2H), 7.66–7.63 (2H), 7.57–7.51 (2H), 7.44–7.37 (8H), 7.33–7.28 (6H), 7.18 and 7.17 (1H in total), 7.10–7.09 (1H), 4.98–4.92 (4H), 4.78–4.75 (4H) (multiplicities and coupling constants could not be determined because of severe overlapping of isomer peaks); ^13^C{^1^H} NMR (101 MHz, CDCl_3_) δ: 154.10, 153.97, 144.85, 144.74, 144.64, 143.79, 143.68, 143.65, 143.39, 143.34, 143.28, 143.24, 143.12, 143.05, 142.94, 142.75, 142.64, 142.57, 142.38, 142.28, 138.59, 137.42, 137.36, 137.23, 137.08, 137.01, 136.92, 135.97, 135.89, 135.83, 135.75, 135.67, 135.58, 135.52, 135.43, 135.37, 135.29, 135.02, 134.94, 133.93, 133.88, 133.80, 133.61, 133.53, 130.98, 130.96, 128.18, 128.14, 127.96, 127.88, 127.83, 127.15, 126.69, 126.64, 126.22, 125.74, 125.68, 125.58, 125.49, 125.02, 124.94, 124.86, 124.81, 124.45, 124.39, 124.09, 124.04, 123.95, 123.91, 123.84, 122.93, 122.39, 122.20, 122.14, 122.10, 121.87, 121.72, 112.24, 112.13, 76.24, 76.13, 47.81, 47.42 (overlapping of signals is expected between stereoisomers); HRMS (MALDI–TOF, DCTB) *m*/*z*: calcd for C_52_H_3_1BrO_6_S_3_, 926.0461 (M^+^); found, 926.0455.

#### 4.2.6. 2,2′-((5-(5-(anthracen-2-yl)thiophen-2-yl)-1,3-phenylene)bis(thiophene-5,2-diyl))bis(9,10- dihydro-9,10-[4,5]epidioxoloanthracen-13-one) (**8**)

Bromide **6** (126 mg, 0.136 mmol), boronate **7** (47.0 mg, 0.155 mmol), K_2_CO_3_ (232 mg, 1.68 mmol), PdCl_2_(dppf)·CH_2_Cl_2_ (16.6 mg, 20.3 μmol, 15 mol%/**6**), and DMF (44 mL) were added to a 100 mL two-necked round-bottom flask. After deoxygenated by argon bubbling for 30 min, the reaction mixture was stirred at 90 °C for 24 h. The mixture was poured into H_2_O (100 mL) and extracted with CH_2_Cl_2_ (3 × 30 mL). The combined organic layer was washed with H_2_O (3 × 30 mL) and brine (30 mL), dried over Na_2_SO_4_, filtered, and evaporated in vacuo. The residue was purified by flash column chromatography on silica gel (CHCl_3_), GPC (CHCl_3_), and reprecipitation (CH_2_Cl_2_/hexanes) to give the target compound as a pale yellow solid (74.9 mg, 73.1 μmol, 54%). The product was obtained as a mixture of stereoisomers regarding the orientation of the carbonate units. ^1^H NMR (400 MHz, CDCl_3_) δ: 8.45 (br s, 1H), 8.42 and 8.41 (1H in total), 8.27 (br s, 1H), 8.06–8.00 (3H), 7.81–7.75 (4H), 7.70 (br s, 2H), 7.57–7.47 (6H), 7.44–7.41 (8H), 7.34–7.27 (6H), 4.98–4.92 (4H), 4.78–4.75 (4H) (multiplicities and coupling constants could not be determined because of severe overlapping of isomer peaks); ^13^C{^1^H} NMR (101 MHz, CDCl_3_) δ: 154.17, 153.99, 144.36, 144.22, 143.50, 143.35, 143.14, 143.00, 142.96, 142.86, 142.59, 142.51, 142.48, 138.53, 138.47, 137.43, 137.36, 137.22, 137.17, 136.94, 136.81, 136.01, 135.92, 135.72, 135.61, 135.51, 135.39, 135.28, 135.16, 133.86, 133.82, 133.62, 133.57, 132.24, 132.21, 131.84, 131.81, 131.59, 130.80, 130.71, 130.66, 128.94, 128.87, 128.22, 128.11, 127.96, 127.87, 127.82, 127.12, 126.68, 126.64, 126.43, 126.22, 126.16, 126.11, 125.75, 125.68, 125.60, 125.53, 124.93, 124.89, 124.80, 124.72, 124.52, 124.49, 124.43, 124.34, 123.72, 123.66, 122.81, 121.70, 121.62, 121.56, 121.47, 76.27, 76.19, 76.13, 47.78, 47.43, 47.38 (overlapping of signals is expected between stereoisomers); HRMS (MALDI–TOF, DCTB) *m*/*z*: calcd for C_66_H_40_O_6_S_3_, 1024.1982 (M^+^); found, 1024.1971.

#### 4.2.7. 2,2′-((5-(5-(anthracen-2-yl)thiophen-2-yl)-1,3-phenylene)bis(thiophene-5,2-diyl))bis(9,10- dihydro-9,10-ethanoanthracene-11,12-diol) (**9**)

Compound **8** (190 mg, 0.185 mmol) and THF (16 mL) were add to a 100 mL round-bottom flask in air. NaOH aq (1.2 M, 16 mL, 19.2 mmol) was added dropwise to the solution, and the mixture was refluxed for 5 h. After cooled down to r.t., the mixture was diluted with H_2_O (20 mL) and extracted with CH_2_Cl_2_ (50 mL; 3 × 20 mL). The combined organic layer was washed with H_2_O (3 × 20 mL) and brine (50 mL), dried over Na_2_SO_4_, filtered, and evaporated in vacuo. The residue was purified by flash column chromatography on silica gel (CH_2_Cl_2_/MeOH, 25:1) to give compound **9** as a pale yellow solid (116 mg, 0.119 mmol, 64%). The product was obtained as a mixture of stereoisomers regarding the orientation of the dihydroxyethylene bridges. ^1^H NMR (400 MHz, CDCl_3_) δ: 8.46 (s, 1H), 8.42 (s, 1H), 8.27 (s, 1H), 8.07–8.01 (3H), 7.81–7.62 (6H), 7.55–7.24, *7.21–7.08 (2H), 4.51–4.47 (4H), 4.18–4.10 (4H), 2.21–2.11 (4H) (multiplicities and coupling constants could not be determined because of severe overlapping of isomer peaks. *Integral value was not determined because of severe overlapping with CHCl_3_); HRMS (MALDI–TOF, DCTB) *m*/*z* calcd for C_64_H_44_O_4_S_3_, 972.2396 (M^+^); found, 972.2391. ^13^C NMR of this compound could not be obtained because of the low solubility. Formation of this compound was indicated by the appearance of the ^1^H NMR peaks at 2.21–2.11 ppm that can be assigned to the hydroxy groups, and the agreement between the expected and observed m/z values in HRMS. More detailed spectral characterization was performed in the next step.

#### 4.2.8. 2,2′-((5-(5-(anthracen-2-yl)thiophen-2-yl)-1,3-phenylene)bis(thiophene-5,2-diyl))bis(9,10-dihydro-9,10-ethanoanthracene-11,12-dione) (TAT(DK)_2_)

To a 50 mL three-necked round-bottom flask containing DMSO (0.30 mL, 0.33 g, 4.2 mmol) and CH_2_Cl_2_ (7 mL) was added dropwise trifluoroacetic anhydride (0.40 mL, 0.60 g, 2.8 mmol) at −78 °C under argon. After stirring for 1 h, a solution of compound **9** (116 mg, 0.119 mmol) in CH_2_Cl_2_ (16 mL) was added dropwise to the mixture. After stirring at the same temperature for 2 h, *N*,*N*-diisopropylethylamine (1.48 mL, 1.10 g, 8.50 mmol) was added dropwise. The mixture was further stirred at −78 °C for 2 h before allowed to warm up to r.t. and stirred for additional 1 h. The reaction was quenched by adding HCl aq (2 M, 6 mL), and the resulting mixture was extracted with CH_2_Cl_2_ (3 × 10 mL). The combined organic layer was washed with H_2_O (3 × 30 mL) and brine (30 mL), dried over Na_2_SO_4_, filtered, and evaporated in vacuo. The crude product was purified by flash column chromatography on silica gel (CH_2_Cl_2_/AcOEt, 50:1) followed by reprecipitation (CH_2_Cl_2_/pentanes) to give TAT(DK)_2_ as a yellow powder (30.9 mg, 32.0 μmol, 27%). ^1^H NMR (400 MHz, CDCl_3_) δ: 8.43 (s, 1H), 8.39 (s, 1H), 8.25 (s, 1H), 7.99–8.04 (3H), * 7.79–7.74 (6H), * 7.65 (dd, *J* = 7.8, 1.8 Hz, 2H), 7.53–7.41 (17H), * 7.35 (d, *J* = 3.7 Hz, 2H), 5.07 (s, 2H), 5.04 (s, 2H) (* Multiplicities and coupling constants could not be determined because of severe peak overlapping); ^13^C{^1^H} NMR (101 MHz, CDCl_3_) δ: 183.62, 183.32, 143.44, 142.70, 135.75, 135.57, 135.37, 134.66, 134.58, 133.90, 132.28, 131.89, 131.63, 130.88, 130.74, 129.60, 129.57, 129.00, 128.25, 128.14, 126.94, 126.68, 126.44, 126.39, 126.25, 125.77, 125.62, 124.95, 124.60, 123.85, 123.82, 123.26, 122.11, 121.94, 60.02, 59.66 (number of peaks is less than expected probably due to overlapping of some aromatic peaks); HRMS (MALDI–TOF, DCTB) *m*/*z*: calcd for C_64_H_36_O_4_S_3_ 964.1770 (M^+^); found 964.1753.

### 4.3. Thin-Film Characterization

UV–vis absorption spectra and Fourier-transform IR spectra were measured on a JASCO V-670 and JASCO FT/IR-4200 spectrometer equipped with an RAS PRO410-H attachment, respectively. The sample films were prepared on glass (for UV−vis) or ITO-glass (for IR) substrate by spin-coating a 10 mg mL^−1^ solution of TAT(DK)_2_ in chloroform at 800 rpm for 30 s. The photoreaction was performed by irradiating with a blue LED (470 ± 10 nm) at 200 mW cm^−2^ for 30 min.

The surface morphology of organic films was analyzed with a Shimadzu SPM-9700 atomic force microscope in the tapping mode using a silicon probe with a resonant frequency of 122 kHz and a force constant of 15 N m^−1^ (SI-DF20). Out-of-plane X-ray diffraction profiles were recorded on a Rigaku RINT-TTR III diffractometer equipped with a rotating anode (Cu-Kα, *λ* = 1.5418 Å) operated at 15 kW and a Rigaku D/teX Ultra 1D silicon strip detector. Measurements were performed in the *θ*–2*θ* mode with a scan range of 2*θ* = 2–30°. The sample films were prepared on ITO-glass substrate in the same manner as the preparation of p-sublayers in OPV fabrication as described in Section 4.4.

The hole-only device for SCLC measurement was fabricated as follows: ITO-patterned glass substrates (20 × 20 mm^2^, 15 Ω per square) were washed by sonicating sequentially in detergent (Furuuchi Chemical Semico Clean 53), distilled water, and isopropanol at r.t. for 10 min each, then subjected to UV–O_3_ treatment (Bioforce Nanoscience, TC-003) for 20 min. On the thus-cleaned substrate, MoO_3_ (15 nm) was vacuum-deposited, and then a TAT layer of 44 nm thickness was prepared by spin-coating of a TAT(DK)_2_ solution in chloroform (800 rpm, 30 s) followed by irradiation with a blue LED (470 ± 10 nm) at 400 mW cm^−2^ for 10 min. MoO_3_ (15 nm) and Al (80 nm) were sequentially vacuum-deposited to complete the device, which was then encapsulated with a glass plate and UV-cure resin in a glove box before measurements for evaluation. The *J*–*V* characteristics of the resulting device were measured in air within the range of 0−10 V using a Keithley 2400 source-measure unit. The current–voltage (*I*–*V*) curve was analyzed based on the Mott–Gurney law describing SCLC:

(1) $$J=\left(8/9\right)\varepsilon_{r}\varepsilon_{0}\mu \left(V^{2}/L^{3}\right)$$

where *J* is the current density (*I*/(device area)), *ε*_r_ is the relative dielectric constant, *ε*_0_ is the permittivity of free space, *μ* is the charge-carrier mobility, *V* is the applied voltage, and *L* is the thickness of the active layer. The dielectric constant *ε*_r_ is assumed to be 3, which is a typical value for organic semiconductors. The active-layer thickness (*L*) was measured using a surface profiler (ET200, Kosaka Laboratory) after the SCLC measurements.

The ionization energies were determined from the onset in photoelectron spectra measured on a Riken Keiki AC-3 photoelectron spectrometer. Here again, the samples were prepared on ITO-glass substrate in the same manner as the preparation of p-sublayers for OPVs but without PEDOT: PSS buffer.

### 4.4. Device Fabrication and Characterization

Indium–tin oxide (ITO)-patterned glass substrates (20 × 20 mm^2^, 15 Ω per square) were cleaned by gentle rubbing with an acetone-soaked wipe for about 5 s, sequential sonication in Semicoclean 56 (Furuuchi Chemical Co.), distilled water, and isopropanol for 10 min each, and then drying with a flow of N_2_ gas. The cleaned substrates were further treated in a UV–O_3_ cleaner (BioForce Nanosciences TC-003) for 20 min, and then PEDOT:PSS (Clevious P VP AI4083) was spin-coated on the substrates at 3000 rpm for 30 s before thermally annealed at 130 °C for 10 min in air. The thickness of the resulting PEDOT:PSS layer was about 25 nm. The substrates were then transferred to a N_2_-filled glovebox (<5 ppm of O_2_ and H_2_O) for preparation of p–i–n-type active layers. The p-sublayer was prepared by spin-coating of TAT(DK)_2_, DTADK, or PhBADTDK (0.5 mg mL^−1^ in CHCl_3_, 800 rpm, 30 s) followed by irradiation with a blue LED (470 nm, 400 mW cm^−2^, 10 min). The i-sublayer was prepared by spin-coating of EBDBTA:PC_71_BM (2:1 by weight) (10 mg mL^−1^ in CHCl_3_, 800 rpm, 30 s) followed by irradiation with a blue LED (470 nm, 400 mW cm^−2^, 30 min). At this point, organic layers were exposed to the vapor of tetrahydrofuran for 60 s at r.t. in a covered Petri dish for annealing. The n-sublayer was then prepared by spin-coating of PC_71_BM (5 mg mL^−1^ in CHCl_3_, 800 rpm, 30 s). The cathode buffer ETL-1 was spin-coated from a 0.5 mg mL^−1^ solution in MeOH (800 rpm, 30 s). Finally, each substrate was transferred to a vacuum-evaporation equipment without exposing to air, and Al (80 nm) was vapor-deposited at ~10^−4^ Pa (10 Å s^−1^) through a shadow mask that defined an active area of 2 × 2 mm^2^. Thus fabricated devices were encapsulated with a glass plate and UV-cure resin in a glove box before measurements for evaluation.

Current-density–voltage (*J*–*V*) curves were measured using a Keithley 2400 source measurement unit under simulated AM 1.5G illumination at an intensity of 100 mW cm^−2^ using a solar simulator (Bunkoukeiki CEP-2000RF).

## Figures and Tables

**Table 1:** Performance of the p–i–n-type OPVs. ^1–4^.

p-Sublayer Material	*J*_SC_/mA cm^−2^	*V*_OC_/V	FF/%	PCE/%	*J*_dark_ (−2 V)/mA cm^−2^
TAT	6.44 (6.30 ± 0.23)	0.899 (0.894 ± 0.005)	55.9 (55.2 ± 1.4)	3.24 (3.11 ± 0.19)	0.18 (0.15 ± 0.02)
DTA	6.23 (6.21 ± 0.14)	0.877 (0.869 ± 0.007)	57.3 (56.1 ± 1.7)	3.13 (3.03 ± 0.14)	0.53 (0.43 ± 0.07)
PhBADT	6.23 (6.12 ± 0.34)	0.876 (0.863 ± 0.011)	57.2 (55.3 ± 1.5)	3.12 (2.93 ± 0.21)	0.54 (0.46 ± 0.11)

^1^*J*_SC_: short-circuit current density, *V*_OC_: open-circuit voltage, FF: fill factor, PCE: power-conversion efficiency, *J*_dark_(−2 V): current-density at −2 V in the dark. ^2^ Values of the best-PCE devices followed by the average and standard deviation of six devices in parentheses. ^3^ Measured under simulated AM1.5G illumination at 100 mW cm^−2^. ^4^ General device structure: (ITO/p-sublayer material/EBDBTA:PC_71_BM/PC_71_BM/ETL-1/Al).

## Supplementary Materials

The following are available online at https://www.mdpi.com/1996-1944/13/10/2316/s1, Figure S1: Results of the DFT computation on TAT performed at the B3LYP/6-31G(d) level of theory: (a) Frontier orbital energies in comparison with those of DTA and PhBADT; (b) Top and side views of the optimized structure. The relevant dihedral angles are shown in the top view, Table S1: Atomic coordinates of the optimized structure of TAT, Figure S2: ^1^H NMR spectrum of compound 2 (CDCl_3_, 400 MHz), Figure S3: ^13^C{^1^H} NMR spectrum of compound 2 (CDCl_3_, 101 MHz), Figure S4: ^1^H NMR spectrum of compound 3 (CDCl_3_, 400 MHz), Figure S5: ^13^C{^1^H} NMR spectrum of compound 3 (CDCl_3_, 101 MHz), Figure S6: ^1^H NMR spectrum of compound 4 (CDCl_3_, 400 MHz), Figure S7: ^13^C{^1^H} NMR spectrum of compound 4 (CDCl_3_, 101 MHz), Figure S8: ^1^H NMR spectrum of compound 5 (CDCl_3_, 400 MHz), Figure S9: ^13^C{^1^H} NMR spectrum of compound 5 (CDCl_3_, 101 MHz), Figure S10: ^1^H NMR spectrum of compound 6 (CDCl_3_, 400 MHz), Figure S11: ^13^C{^1^H} NMR spectrum of compound 6 (CDCl_3_, 101 MHz), Figure S12: ^1^H NMR spectrum of compound 8 (CDCl_3_, 400 MHz), Figure S13: ^13^C{^1^H} NMR spectrum of compound 8 (CDCl_3_, 101 MHz), Figure S14: ^1^H NMR spectrum of compound 9 (CDCl_3_, 400 MHz), Figure S15: ^1^H NMR spectrum of TAT(DK)_2_ (CDCl_3_, 400 MHz), Figure S16: ^13^C{^1^H} NMR spectra of TAT(DK)_2_ (CDCl_3_, 101 MHz), Figure S17: Tapping-mode AFM images of thin films prepared through the photoprecursor approach: (a) TAT (*R*_RMS_ = 1.0 nm); (b) PhBADT (12.4 nm). The scale bars correspond to 1.0 μm. See references 16 and 21 for additional AFM images of DTA, Figure S18: *J*–*V* curve of the hole-only device with EBDBTA. Voltage for the observed data is defined as *V*_appl_–*V*_bi_–*V*_s_, wherein *V*_appl_ is the applied voltage, *V*_bi_ is the estimated built-in voltage, and *V*_s_ is the estimated voltage drop associated with series resistance, Figure S19: Photoelectron spectrum of TAT deposited via the photoprecursor approach on ITO substrate, Figure S20: Semi-log *J*–*V* plots for the p–i–n-type OPVs comprising different p-sublayer materials: (a) TAT; (b) DTA; (c) PhBADT. The data are the same as those plotted in Figure 8 in the main text.
