# Supplementary material for: A Windmill-Shaped Molecule with Anthryl Blades to Form Smooth Hole-Transport Layers via a Photoprecursor Approach
Source: Materials (Basel). 2020 May 18;13(10):2316. doi: 10.3390/ma13102316 (PMC7287758; doi:10.3390/ma13102316)
Supplement: Supplementary file 1 [file materials-13-02316-s001.pdf]

materials

Supplementary Materials

A Windmill-Shaped Molecule with Anthryl Blades to Form Smooth Hole-Transport Layers via a Photoprecursor Approach

Akihiro Maeda [1], Aki Nakauchi [1], Yusuke Shimizu [1], Kengo Terai [1], Shuhei Sugii [1], Hironobu Hayashi [1], Naoki Aratani [1], Mitsuharu Suzuki [2,*] and Hiroko Yamada [1,*]

[1] Division of Materials Science, Graduate School of Science and Technology, Nara Institute of Science and Technology (NAIST), 8916-5 Takayama-cho, Ikoma, Nara 630-0192, Japan; wsgtx310@gmail.com (A.M.); masquarade.ribbon@icloud.com (A.N.); orgvy3s92@gmail.com (Y.S.); me1307kengo@gmail.com (K.T.); sshuhei.0627@gmail.com (S.S.); hhayashi@ms.naist.jp (H.H.); aratani@ms.naist.jp (N.A.)

[2] Division of Applied Chemistry, Graduate School of Engineering, Osaka University, 2-1 Yamadaoka, Suita, Osaka 565-0871, Japan

* Correspondence: msuzuki@chem.eng.osaka-u.ac.jp (M.S.); hyamada@ms.naist.jp (H.Y.)

1. Computation

The structure of TAT was optimized at the B3LYP/6-31G(d) level of theory with a C_3-symmetry constraint. The estimated HOMO and LUMO energies are −5.14 and −1.93 eV; thus, the HOMO of TAT is somewhat stabilized as compared to those of the previously employed p-sublayer materials DTA and PhBADT (Figure S1a). In terms of the HOMO–LUMO energy difference, TAT ($\Delta E_{HOMO–LUMO}$ = 3.21 eV) was calculated to be in between DTA (3.18 eV) and PhBADT (3.29 eV). The optimized conformation is rather planar with dihedral angles below 30° (Figure S1b, Table S1), which is favorable for forming π–π stacking in the thin-film state.

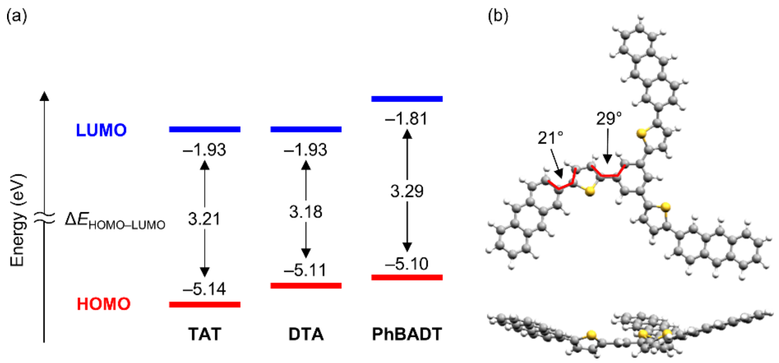

Figure S1. Results of the DFT computation on TAT performed at the B3LYP/6-31G(d) level of theory: (**a**) Frontier orbital energies in comparison with those of DTA and PhBADT; (**b**) Top and side views of the optimized structure. The relevant dihedral angles are shown in the top view.

Table S1. Atomic coordinates of the optimized structure of TAT.

Atom No.	Symbol	X	Y	Z	Atom No.	Symbol	X	Y	Z
1	C	1.193	0.723	0.960	51	C	4.725	−5.430	0.121
2	C	−0.029	1.411	0.964	52	C	5.539	−6.557	−0.193
3	C	−1.222	0.671	0.960	53	C	6.973	−6.392	−0.233
4	C	−1.208	−0.731	0.964	54	C	7.508	−5.094	0.038
5	C	0.030	−1.394	0.960	55	C	6.694	−4.035	0.325
6	C	1.237	−0.681	0.964	56	C	4.993	−7.820	−0.458

7	C	2.533	−1.371	0.975	57	C	5.801	−8.924	−0.762
8	C	−0.079	2.878	0.975	58	C	7.236	−8.756	−0.801
9	C	−2.453	−1.508	0.975	59	C	7.781	−7.492	−0.535
10	C	−1.054	3.697	1.501	60	C	5.255	−10.217	−1.036
11	C	−0.786	5.081	1.344	61	C	6.071	−11.278	−1.330
12	C	0.402	5.350	0.697	62	C	7.486	−11.112	−1.369
13	S	1.191	3.847	0.255	63	C	8.049	−9.889	−1.112
14	C	−2.674	−2.762	1.501	64	H	2.123	1.283	0.952
15	C	−4.007	−3.221	1.344	65	H	−2.172	1.197	0.952
16	C	−4.834	−2.327	0.697	66	H	0.050	−2.479	0.952
17	S	−3.927	−0.892	0.255	67	H	−1.926	3.314	2.021
18	C	3.729	−0.935	1.501	68	H	−1.435	5.857	1.733
19	C	4.793	−1.860	1.344	69	H	−1.907	−3.325	2.021
20	C	4.432	−3.023	0.697	70	H	−4.355	−4.172	1.733
21	S	2.736	−2.955	0.255	71	H	3.833	0.011	2.021
22	C	5.264	−4.185	0.377	72	H	5.790	−1.685	1.733
23	C	−6.256	−2.466	0.377	73	H	3.008	5.950	0.173
24	C	0.992	6.651	0.377	74	H	0.000	9.914	−0.002
25	C	2.340	6.807	0.121	75	H	−0.918	7.699	0.496
26	C	2.909	8.075	−0.193	76	H	4.926	7.362	−0.428
27	C	2.049	9.235	−0.233	77	H	1.947	11.356	−0.565
28	C	0.657	9.049	0.038	78	H	6.868	8.786	−1.006
29	C	0.148	7.815	0.325	79	H	7.792	11.013	−1.537
30	C	4.276	8.234	−0.458	80	H	3.888	12.786	−1.141
31	C	4.828	9.486	−0.762	81	H	−6.656	−0.370	0.173
32	C	3.965	10.644	−0.801	82	H	−8.586	−4.957	−0.002
33	C	2.598	10.485	−0.535	83	H	−6.209	−4.644	0.496
34	C	6.220	9.660	−1.036	84	H	−8.839	0.585	−0.428
35	C	6.731	10.896	−1.330	85	H	−10.808	−3.992	−0.565
36	C	5.880	12.039	−1.369	86	H	−11.043	1.554	−1.006
37	C	4.540	11.915	−1.112	87	H	−13.434	1.241	−1.537
38	C	−7.065	−1.377	0.121	88	H	−13.017	−3.026	−1.141
39	C	−8.448	−1.518	−0.193	89	H	3.649	−5.579	0.173
40	C	−9.022	−2.843	−0.233	90	H	8.586	−4.957	−0.002
41	C	−8.165	−3.955	0.038	91	H	7.127	−3.055	0.496
42	C	−6.842	−3.780	0.325	92	H	3.913	−7.947	−0.428
43	C	−9.269	−0.414	−0.458	93	H	8.861	−7.364	−0.565
44	C	−10.629	−0.562	−0.762	94	H	4.175	−10.341	−1.006
45	C	−11.201	−1.888	−0.801	95	H	5.642	−12.254	−1.537
46	C	−10.379	−2.992	−0.535	96	H	9.129	−9.760	−1.141
47	C	−11.475	0.557	−1.036	97	H	8.117	−11.965	−1.604
48	C	−12.802	0.381	−1.330	98	H	6.303	13.012	−1.604
49	C	−13.366	−0.927	−1.369	99	H	−14.420	−1.047	−1.604
50	C	−12.589	−2.026	−1.112	–	–	–	–	–

2. NMR spectra of the photoprecursor TAT(DK)$_2$

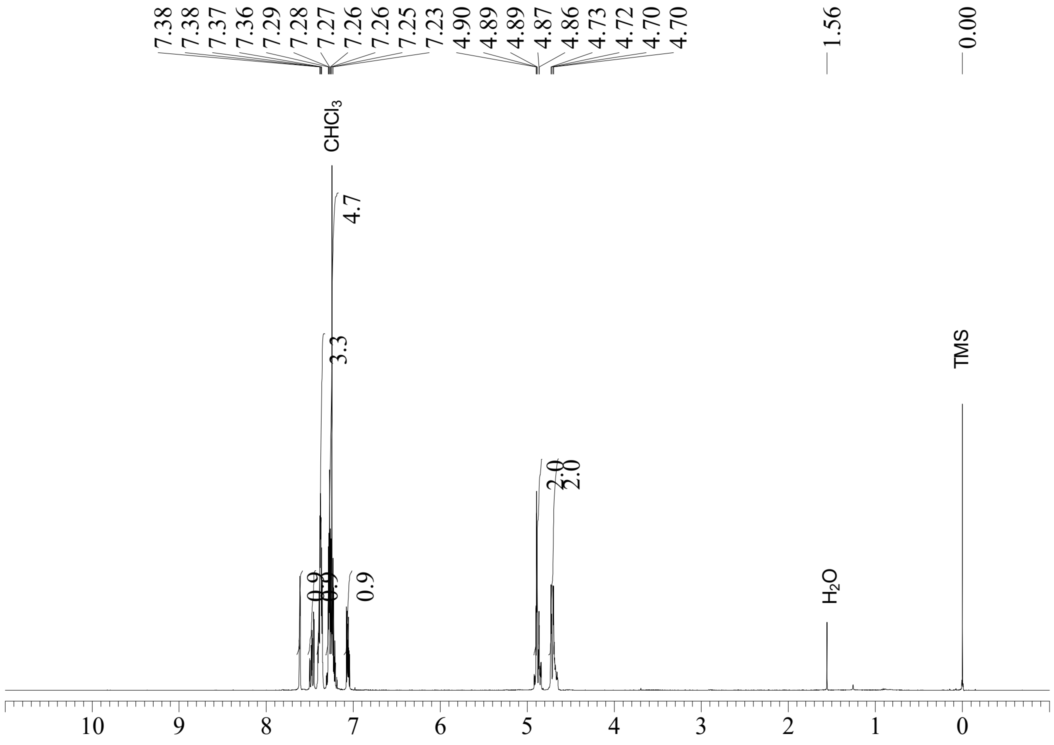

Figure S2. ^{1}H NMR spectrum of compound **2** ($CDCl_3$, 400 MHz).

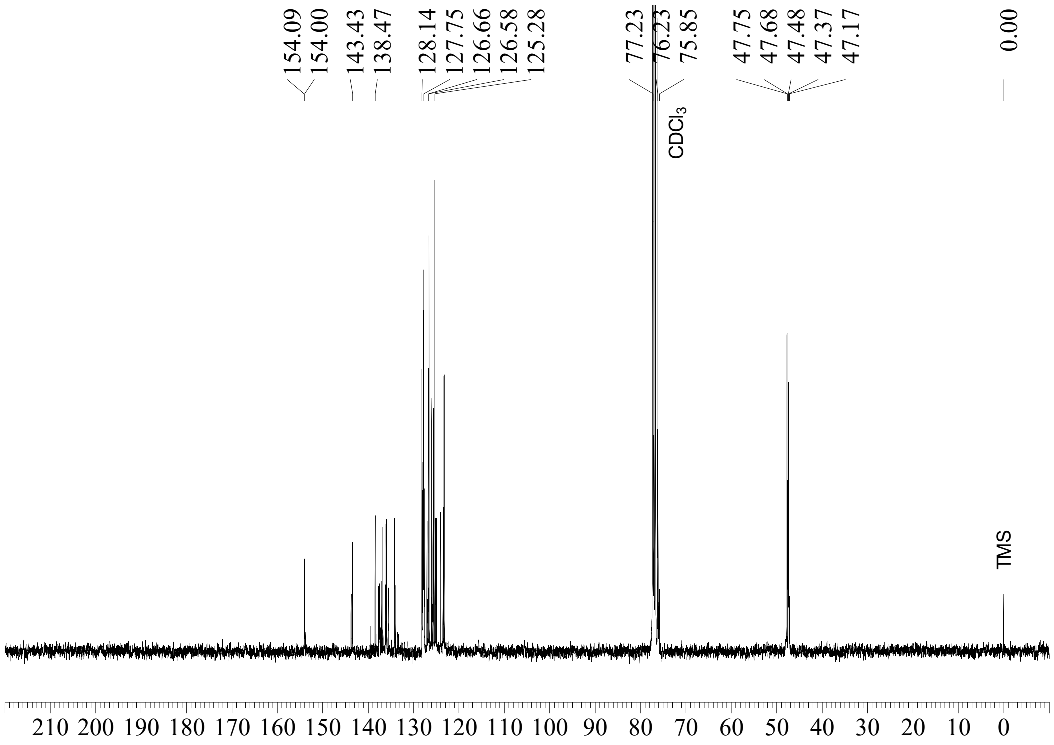

Figure S3. $^{13}C\{^{1}H\}$ NMR spectrum of compound **2** ($CDCl_3$, 101 MHz).

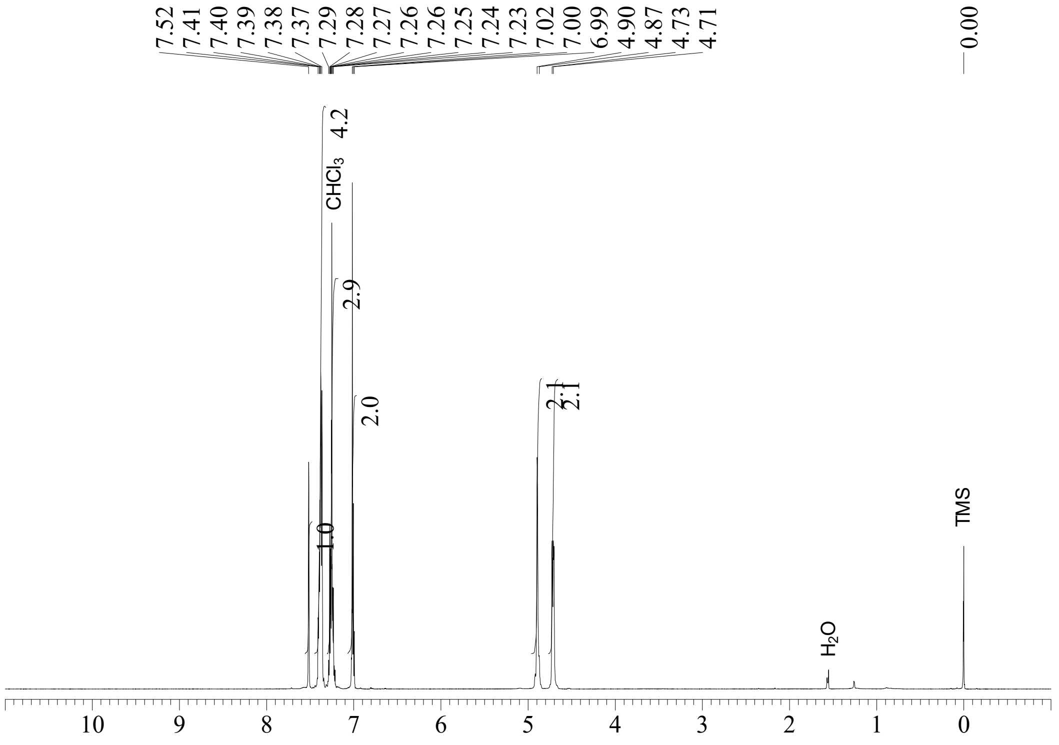

Figure S4. ^{1}H NMR spectrum of compound **3** ($CDCl_3$, 400 MHz).

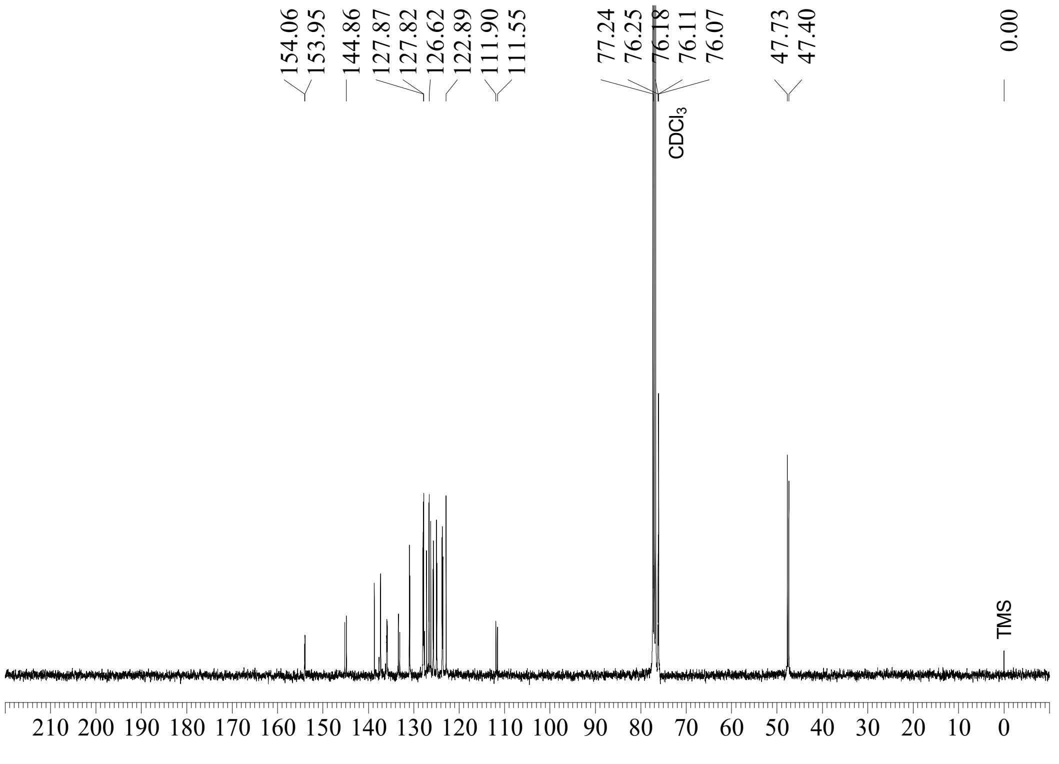

Figure S5. $^{13}C\{^{1}H\}$ NMR spectrum of compound **3** ($CDCl_3$, 101 MHz).

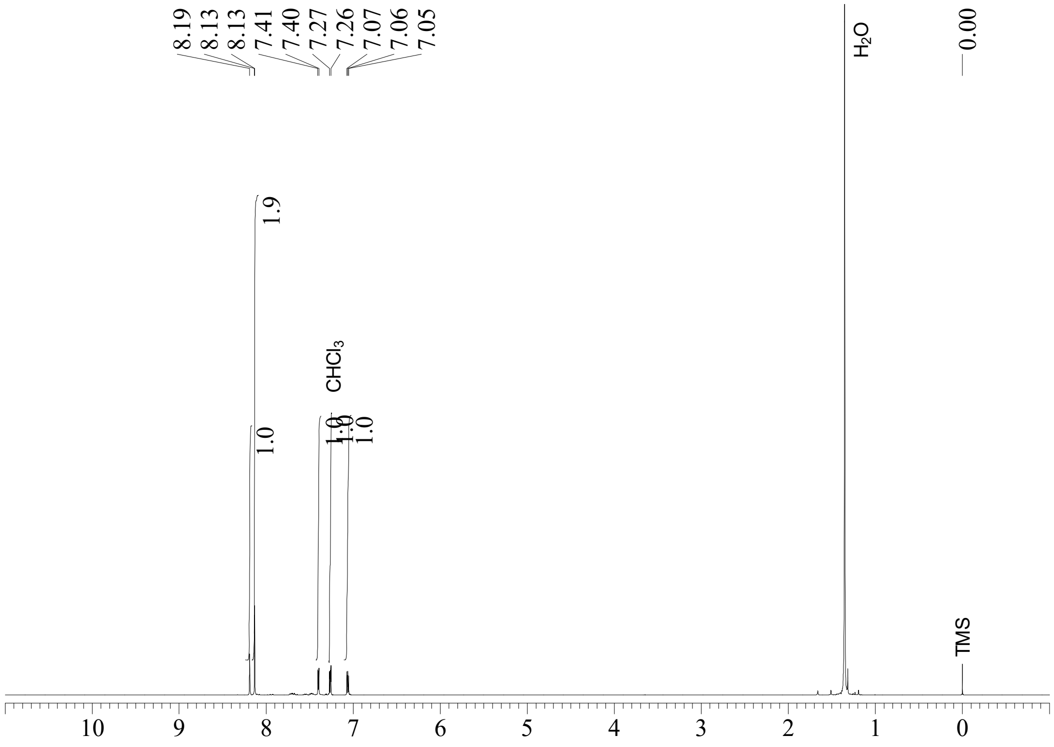

Figure S6. ^{1}H NMR spectrum of compound **4** ($CDCl_3$, 400 MHz).

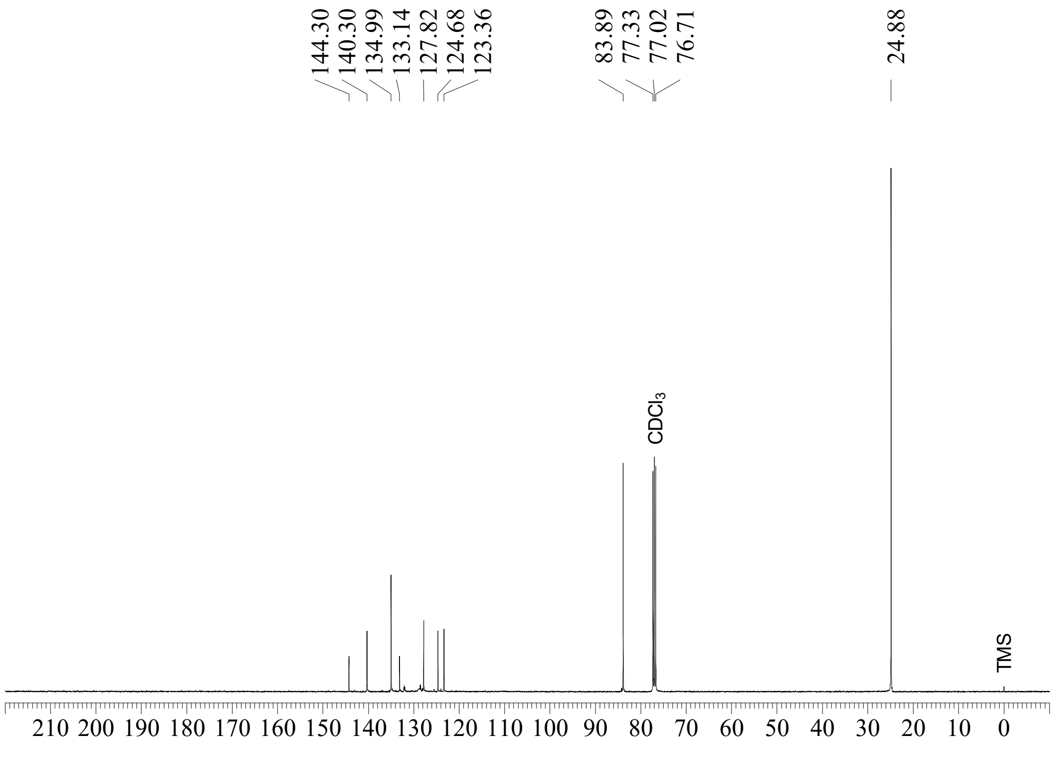

Figure S7. ^{13}C{^{1}H} NMR spectrum of compound **4** ($CDCl_3$, 101 MHz).

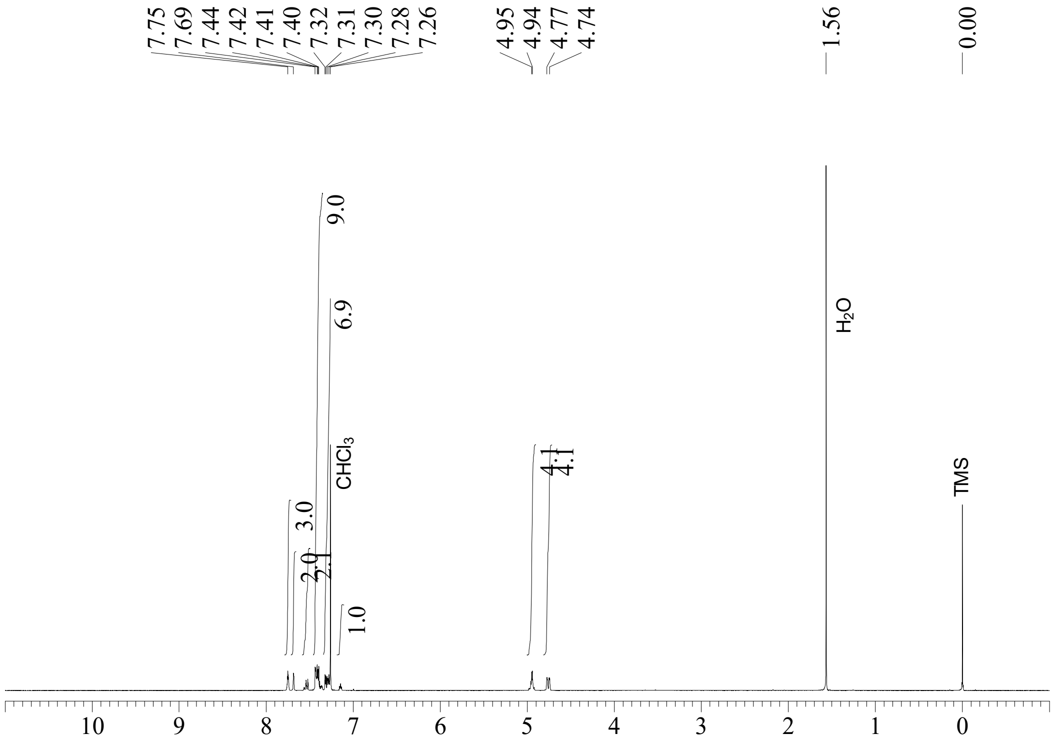

Figure S8. ^{1}H NMR spectrum of compound **5** ($CDCl_3$, 400 MHz).

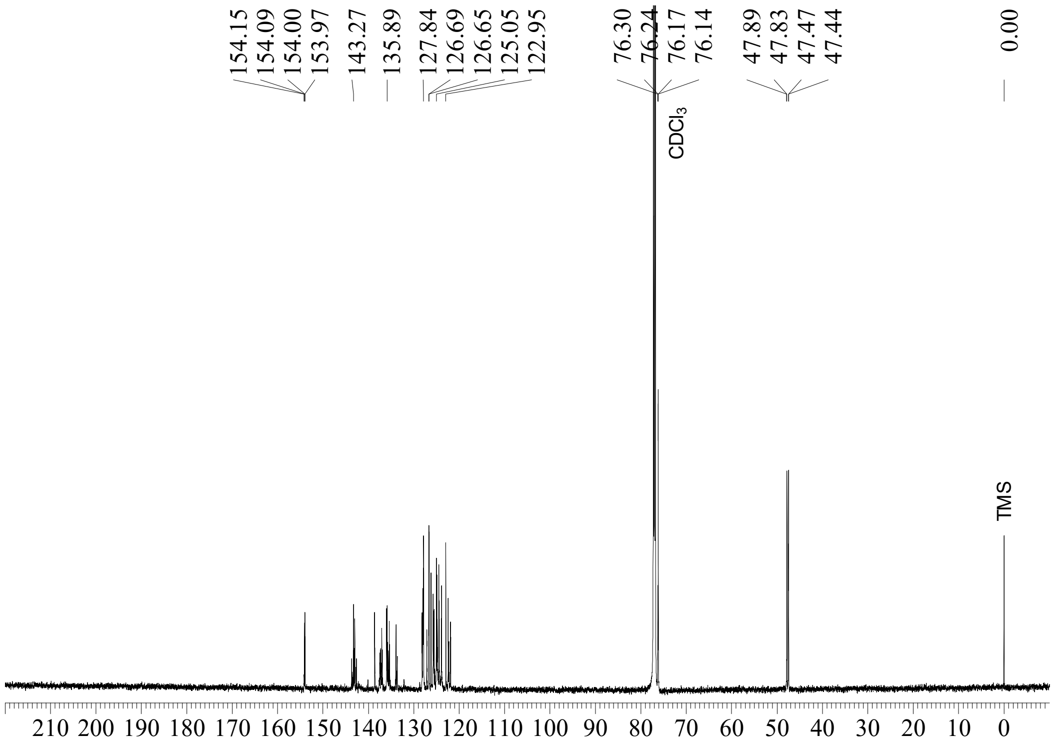

Figure S9. $^{13}C\{^{1}H\}$ NMR spectrum of compound **5** ($CDCl_3$, 101 MHz).

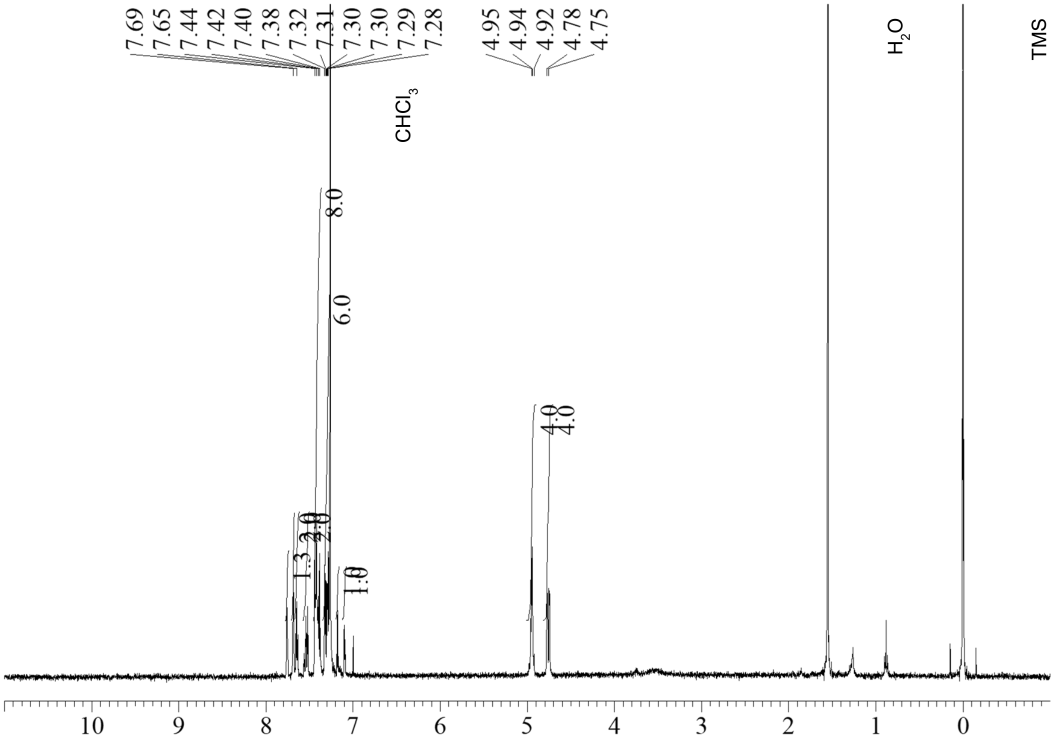

Figure S10. ^{1}H NMR spectrum of compound **6** ($CDCl_3$, 400 MHz).

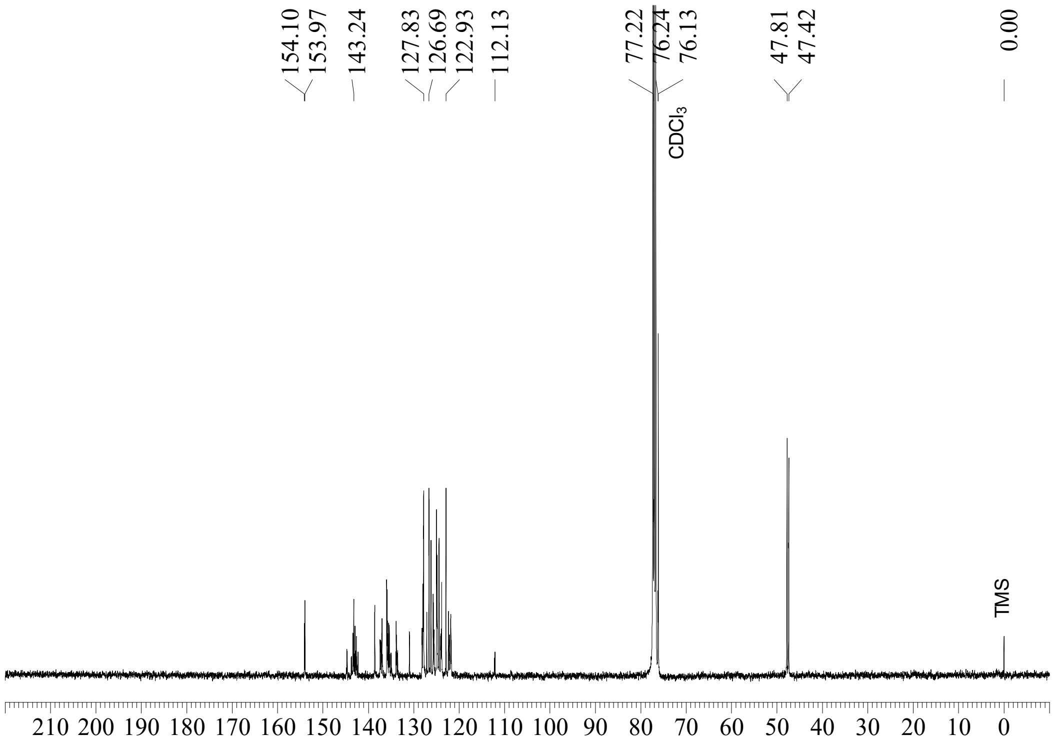

Figure S11. $^{13}C\{^{1}H\}$ NMR spectrum of compound **6** ($CDCl_3$, 101 MHz).

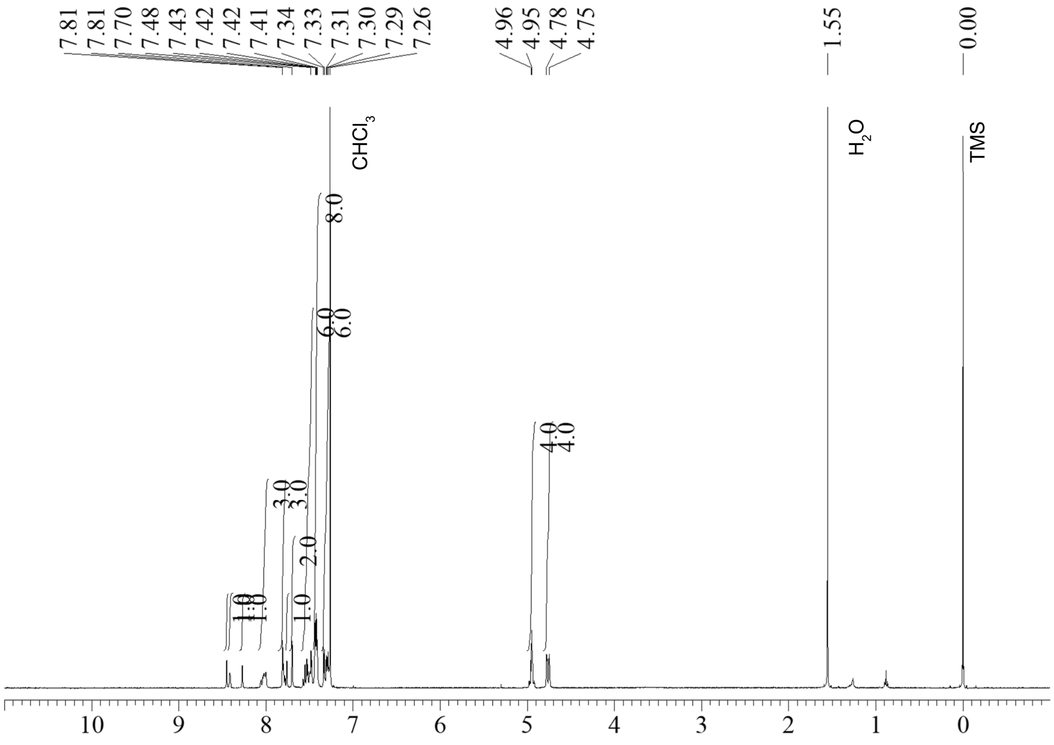

Figure S12. ^{1}H NMR spectrum of compound **8** ($CDCl_3$, 400 MHz).

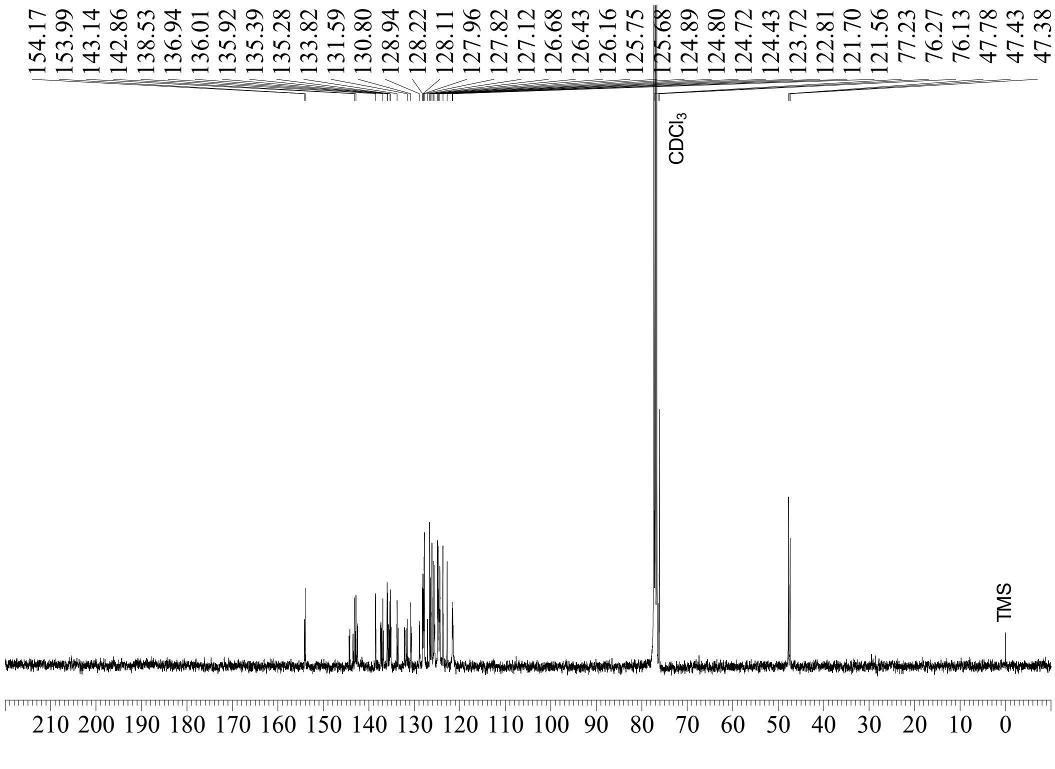

Figure S13. $^{13}C\{^{1}H\}$ NMR spectrum of compound **8** ($CDCl_3$, 101 MHz).

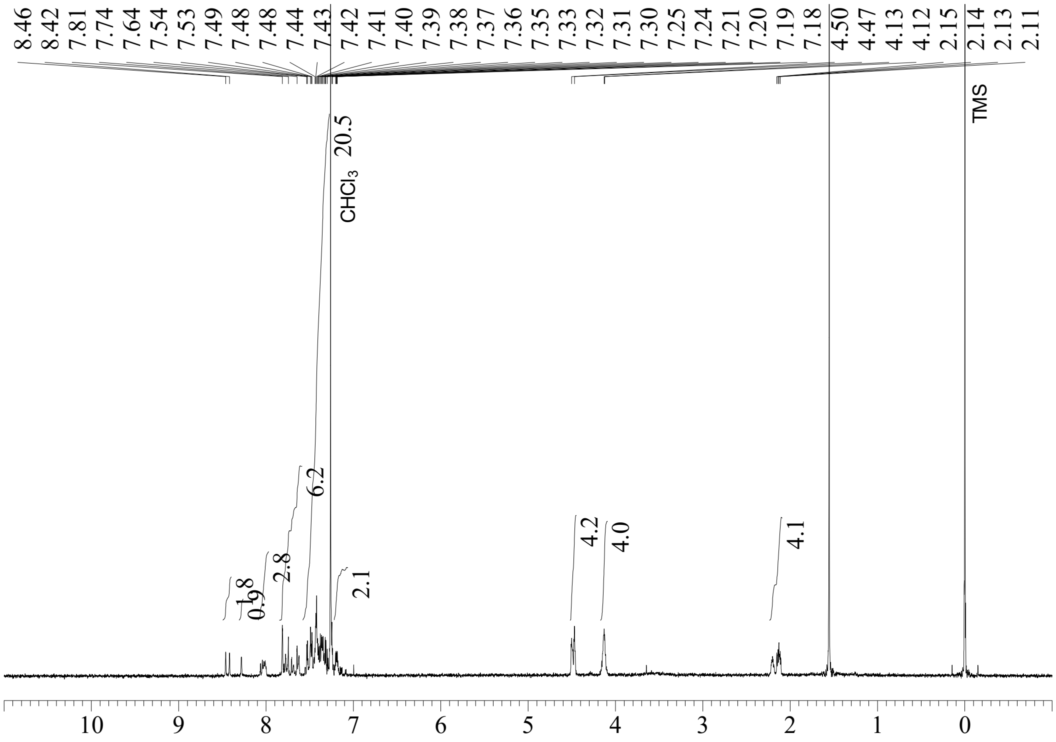

Figure S14. ^{1}H NMR spectrum of compound **9** ($CDCl_3$, 400 MHz).

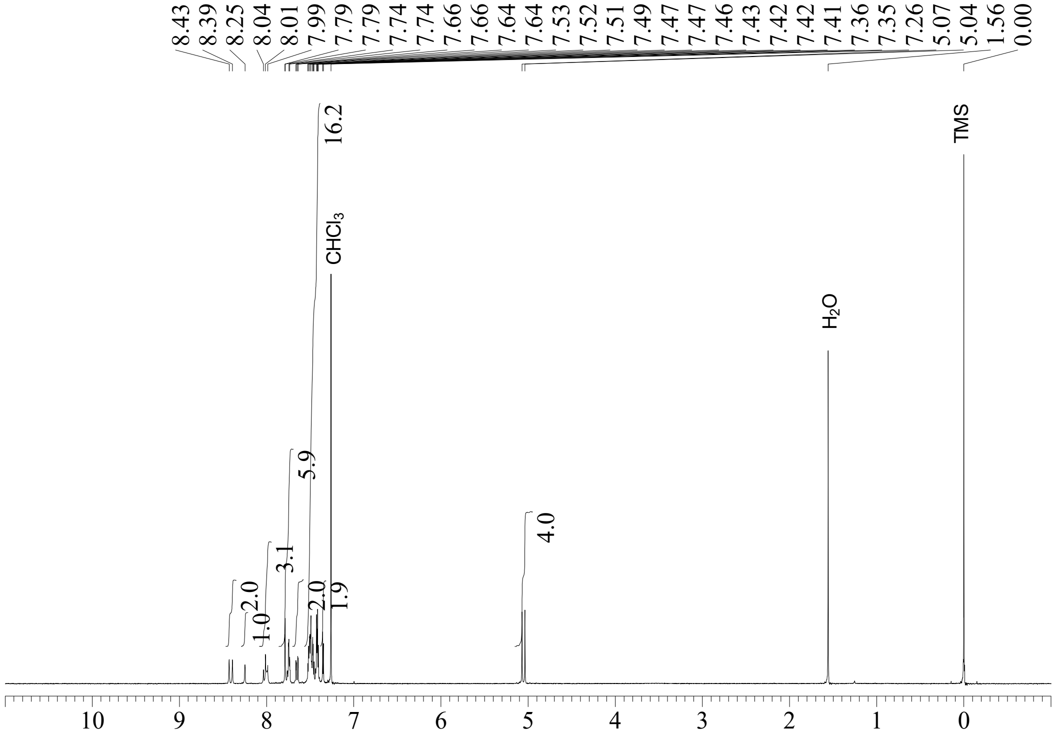

Figure S15. ^{1}H NMR spectrum of TAT(DK)$_2$ ($CDCl_3$, 400 MHz).

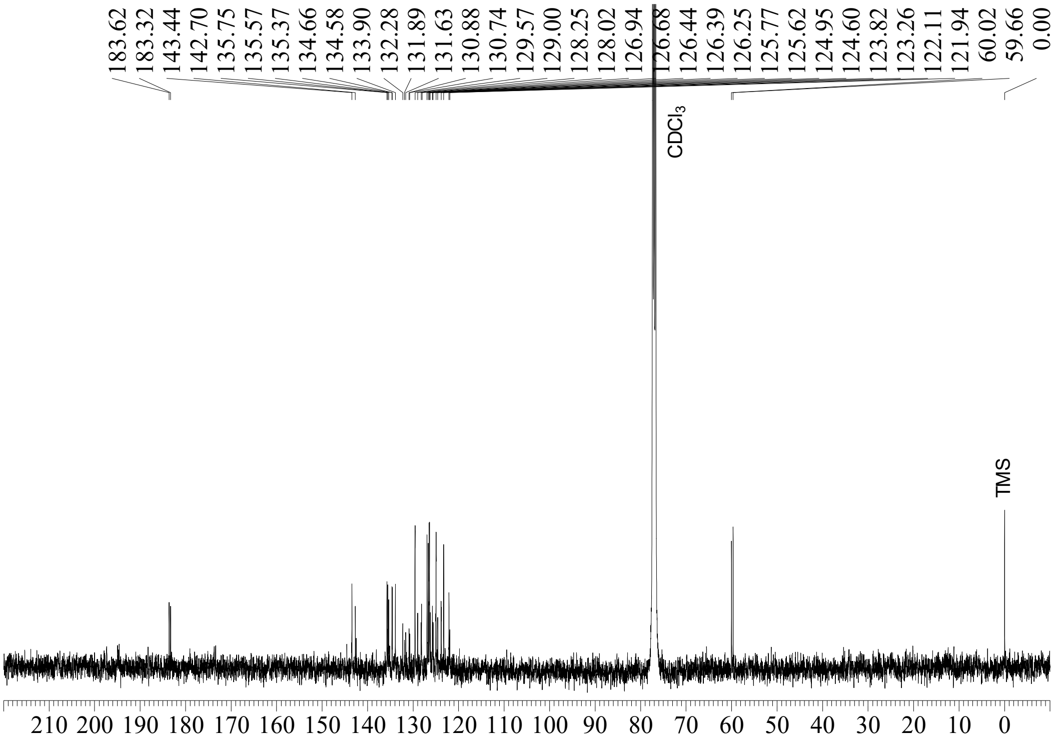

Figure S16. $^{13}C\{^1H\}$ NMR spectra of $TAT(DK)_2$ ($CDCl_3$, 101 MHz).

3. Additional Atomic-Force-Microscopy Images

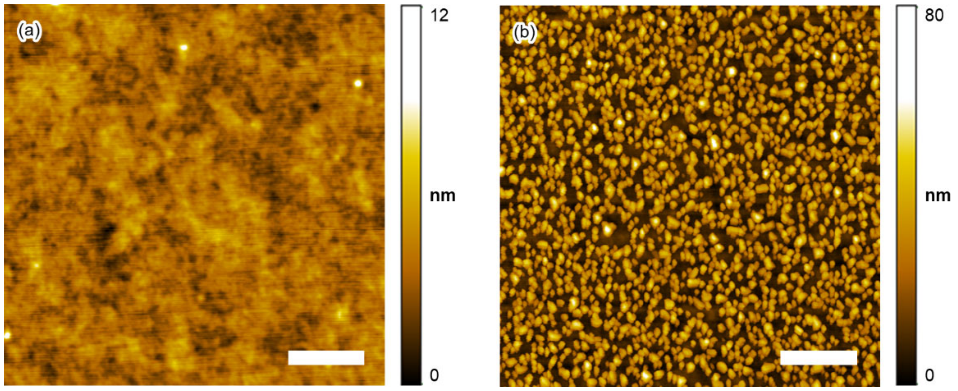

Figure S17. Tapping-mode AFM images of thin films prepared through the photoprecursor approach: (**a**) TAT (R_{RMS} = 1.0 nm); (**b**) PhBADT (12.4 nm). The scale bars correspond to 1.0 μm. See references 16 and 21 for additional AFM images of DTA.

4. Hole Mobility in EBDBTA

The hole mobility in EBDBTA was estimated by the SCLC method in a hole-only device with a structure of [ITO/MoO_3 (10 nm)/EBDBTA (75 nm)/MoO_3 (10 nm)/Al (80 mn)]. The EBDBTA was deposited through the photoprecursor approach as described in a previous paper [1]. The hole mobility was estimated as 6.4×10^{-5} cm^2 V^{-1} s^{-1}.

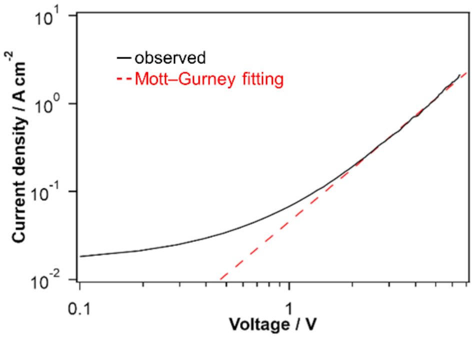

Figure S18. *J*–*V* curve of the hole-only device with EBDBTA. Voltage for the observed data is defined as V_{appl}–V_{bi}–V_s, wherein V_{appl} is the applied voltage, V_{bi} is the estimated built-in voltage, and V_s is the estimated voltage drop associated with series resistance.

5. Photoelectron Spectroscopy of TAT

The ionization energy of EBDBTA was determined as 5.6 eV by photoelectron spectroscopy in air as described in Section 4 of the main text.

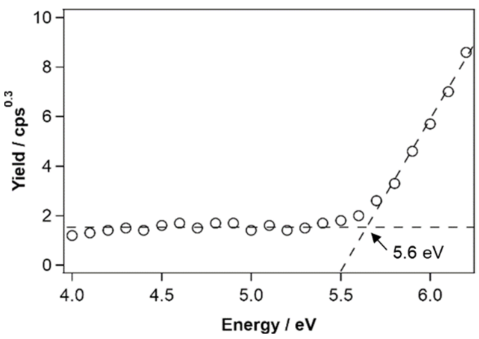

Figure S19. Photoelectron spectrum of TAT deposited via the photoprecursor approach on ITO substrate.

6. Semi-Log Current-Density–Voltage Plots of the p–i–n Devices

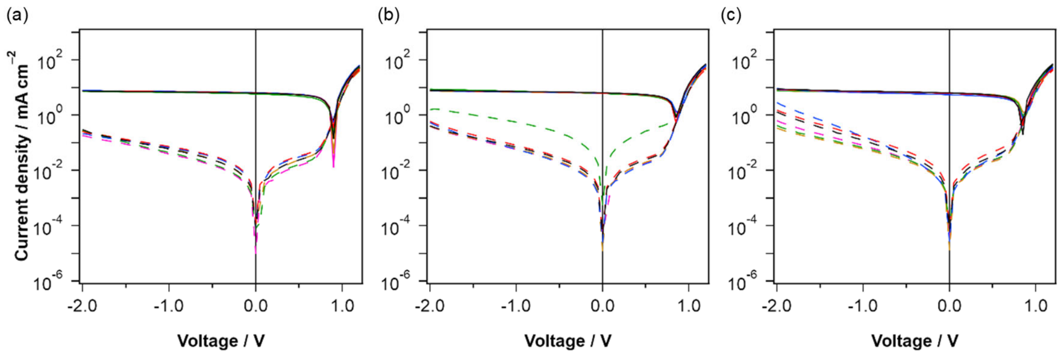

Figure S20. Semi-log *J*–*V* plots for the p–i–n-type OPVs comprising different p-sublayer materials: (**a**) TAT; (**b**) DTA; (**c**) PhBADT. The data are the same as those plotted in Figure 8 in the main text.

Reference

1. Suzuki, M.; Terai, K.; Quinton, C.; Hayashi, H.; Aratani, N.; Yamada, H. Open-circuit-voltage shift of over 0.5 V in organic photovoltaic cells induced by a minor structural difference in alkyl substituents. *Chem. Sci.* **2020**, *11*, 1825–1831.

© 2020 by the authors. Submitted for possible open access publication under the terms and conditions of the Creative Commons Attribution (CC BY) license (http://creativecommons.org/licenses/by/4.0/).
